# Understanding marine larval dispersal in a broadcast-spawning invertebrate: A dispersal modelling approach for optimising spat collection of the Fijian black-lip pearl oyster *Pinctada margaritifera*

**DOI:** 10.1371/journal.pone.0234605

**Published:** 2020-06-18

**Authors:** Monal M. Lal, Cyprien Bosserelle, Pranesh Kishore, Paul C. Southgate

**Affiliations:** 1 Australian Centre for Pacific Islands Research, School of Science and Engineering, University of the Sunshine Coast, Maroochydore, Queensland, Australia; 2 School of Marine Studies, Faculty of Science, Technology and Environment, University of the South Pacific, Suva, Fiji Islands; 3 National Institute of Water and Atmospheric Research, Christchurch, New Zealand; 4 Geoscience, Energy and Maritime Division, Pacific Community (SPC), Nabua, Suva, Fiji Islands; Department of Agriculture, Water and the Environment, AUSTRALIA

## Abstract

Fisheries and aquaculture industries worldwide remain reliant on seed supply from wild populations, with their success and sustainability dependent on consistent larval recruitment. Larval dispersal and recruitment in the marine environment are complex processes, influenced by a multitude of physical and biological factors. Biophysical modelling has increasingly been used to investigate dispersal and recruitment dynamics, for optimising management of fisheries and aquaculture resources. In the Fiji Islands, culture of the black-lip pearl oyster (*Pinctada margaritifera*) is almost exclusively reliant on wild-caught juvenile oysters (spat), through a national spat collection programme. This study used a simple Lagrangian particle dispersal model to investigate current-driven larval dispersal patterns, identify potential larval settlement areas and compare simulated with physical spat-fall, to inform targeted spat collection efforts. Simulations successfully identified country-wide patterns of potential larval dispersal and settlement from 2012–2015, with east-west variations between bi-annual spawning peaks and circulation associated with El Niño Southern Oscillation. Localised regions of larval aggregation were also identified and compared to physical spat-fall recorded at 28 spat collector deployment locations. Significant and positive correlations at these sites across three separate spawning seasons (*r*(26) = 0.435; *r*(26) = 0.438; *r*(26) = 0.428 respectively, *p* = 0.02), suggest high utility of the model despite its simplicity, for informing future spat collector deployment. Simulation results will further optimise black-lip pearl oyster spat collection activity in Fiji by informing targeted collector deployments, while the model provides a versatile and highly informative toolset for the fishery management and aquaculture of other marine taxa with similar life histories.

## Introduction

Ocean currents are a key physical feature of the marine environment, influencing species' diversity, distribution, reproduction and abundance [1]. Considering the vast majority of marine organisms are broadcast spawners with pelagic larval dispersal [2,3], ocean current dynamics directly impact their population connectivity, recruitment patterns, stock and population genetic structures, physiology, morphology and behaviour [4–6]. Developing an understanding of how currents and other oceanographic factors influence key life-history traits of marine organisms is vital for their conservation and management, particularly for species that are important fisheries or aquaculture resources [7].

Globally, many fisheries and aquaculture operations remain reliant on wild populations, and their sustainability and success depend on consistent recruitment and supply of juvenile and or adult individuals [5,8]. Larval dispersal and recruitment dynamics in the marine environment are complex, and governed by a multitude of physical, biological and behavioural variables including, but not limited to, source and sink location bathymetry, gamete release locations (pelagic vs. benthic species), passive dispersal vs. active larval swimming behaviour, pelagic larval duration (PLD), fecundity, larval survival, prevailing current regimes and larval homing abilities [7,9–11]. Due to these factors, measuring larval dispersal, development and settlement during field studies presents many challenges, and therefore the development of biophysical models has become increasingly important to understand larval transport and settlement pathways [11].

Biophysical models typically incorporate biological and physical information, and seek to identify dispersal pathways between larval source (spawning) and sink (recruitment) sites, along with transport corridors between them [7]. Models have also been used to support population genetic analyses to determine connectivity [6,12,13], evaluate biological factors affecting larval dispersal [7,10,14] and develop dispersal models for particular taxa [9,15,16]. For sedentary benthic taxa such as bivalve molluscs, stocks may occupy a discrete geographic region as large as an entire reef system, or as small as a single bivalve bed [5]. When coupled with the highly variable settlement rates that are characteristic of many bivalves [17,18], the survival or extirpation of local populations or whole stocks is entirely dependent on larval recruitment.

Knowledge of larval dispersal pathways, recruitment sites and seasonality is vital for effectively managing increasing fishing pressure, and aquaculture production demands on global bivalve resources [4,5]. Once available, such information may be used to delineate stock boundaries, assist stock recovery actions and perform assessments of recruitment success [5,12,19]. The application of biophysical modelling in these contexts can offer powerful insights into past, present and future recruitment and dispersal patterns, for conservation and management of bivalves, and other marine taxa [11,20].

In the Fiji Islands, the black-lip pearl oyster *Pinctada margaritifera* is the basis of a valuable aquaculture industry that is almost exclusively reliant on oysters collected from the wild [12,21]. The industry has developed and diversified over the past two decades, however a major impediment to increasing productivity has been an inconsistent supply of juvenile oysters (called spat) to farmers. To address this bottleneck, a national spat collection programme has been developed, to improve spat supply reliability [22]. A recent study by Kishore et al. [22] evaluated twenty-eight sites across the Fiji Islands using standard spat collection equipment and methodology [21], to identify sites where *P*. *margaritifera* recruits in large numbers, against locations where lower settlement rates are observed. Based on these data, the Fijian national spat collection program is now able to focus only on high yielding sites, to improve spat availability to pearl farmers in the country [21,22].

The aim of this study was to utilise dispersal modelling to provide additional data for Fiji's national *P*. *margaritifera* spat collection programme. Larval dispersal was modelled over four years from 2012–2015, and compared to actual spat recruitment data recorded at twenty-eight sites in Fiji by Kishore et al. [22]. The dispersal model used was modified from a hydrodynamic particle dispersal model described in earlier studies by Lal et al. [12] and Lal et al. [6], which examined the population genetic structure and connectivity of *P*. *margaritifera*. Data generated by model simulations were used to identify putative current-driven dispersal and potential recruitment patterns of black-lip pearl oyster larvae in Fiji, to inform the country's national spat collection programme. Specifically, this study sought to identify prevailing current-driven dispersal flux patterns in Fiji, identify potential larval aggregation/spat settlement areas, and compare simulated particle count data with actual *P*. *margaritifera* spatfall at the twenty-eight spat collector deployment sites reported by Kishore et al. [22]. This information will further optimise black-lip pearl oyster spat collection efforts in Fiji by informing targeted collector deployments, and has high utility for fishery management and aquaculture of other marine taxa with similar life histories.

## Materials and methods

### Study location

**Fig 1:**
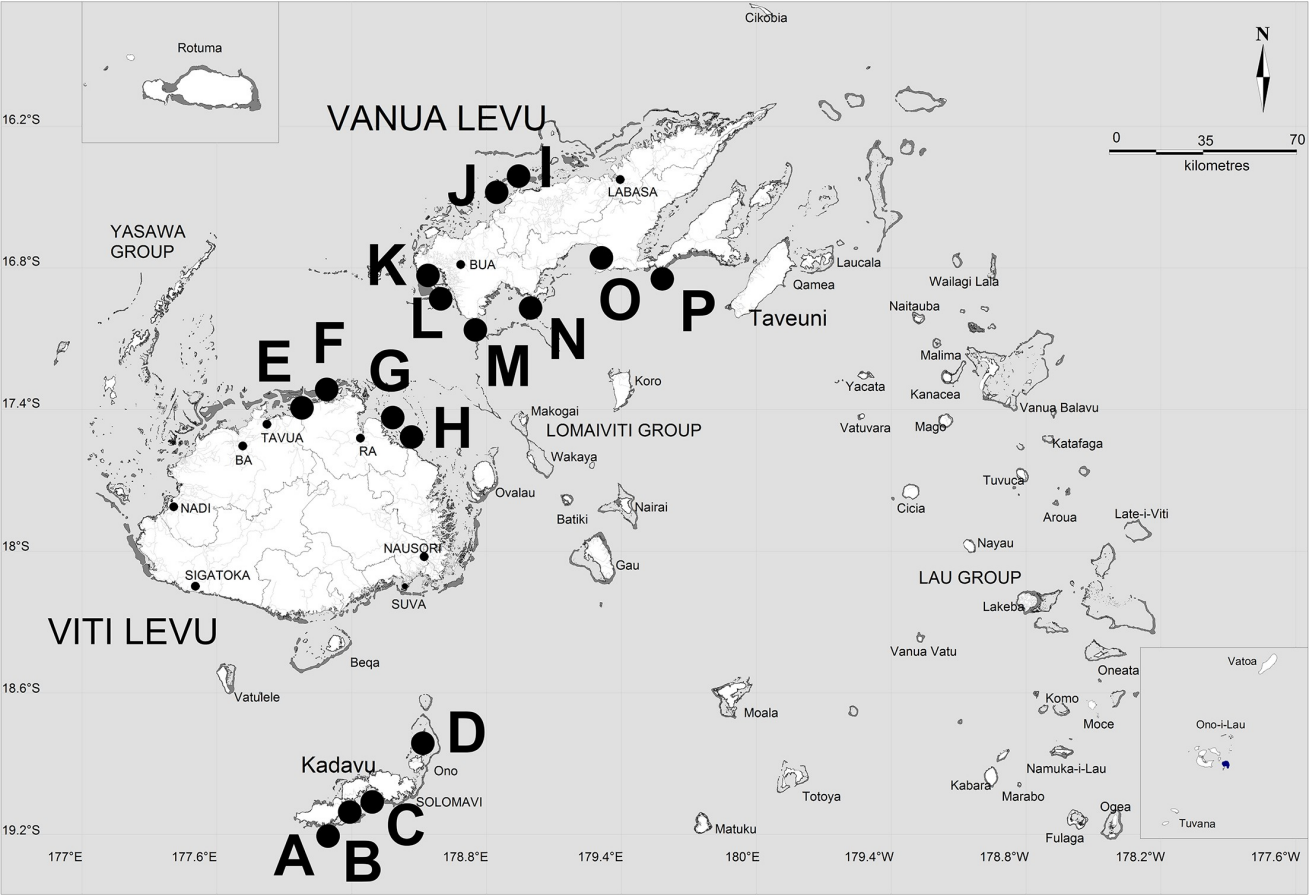
Map of study area in the Fiji Islands adapted from Lal et al. [12]. Reef outlines are presented in dark grey. Site annotations depict spat collector deployment locations studied by Kishore et al. [22]. Collector deployment sites were as follows: Ravitaki (**A**), Galoa (**B**), Dravuwalu (**C**), Naqara (**D**), Vitawa (**E**), Malake A, B, C, D, E and F (**F**), Nacobau (**G**), Namarai A and B (**H**), Raviravi C (**I**), Raviravi A and B (**J**), Tavulomo A, B and C (**K**), Tavulomo D (**L**), Vuya (**M**), Navatu A and B (**N**), Urata (O) and Naweni A and B (**P**). The island of Rotuma and archipelago of Ono i Lau are shown inset.

The Republic of the Fiji Islands is a group of over 330 volcanic islands located in the Southwest Pacific Ocean (**Fig 1**), between the latitudes of 15° to 22° S and longitudes 177° W to 174° E. The country occupies a total land area of 18,333 Km^2^, dispersed across approximately 1,282,980 Km^2^ of ocean surface area [23]. Fiji possesses an oceanic tropical marine climate, with major influences from the El Niño Southern Oscillation (ENSO) circulation, the South Pacific Convergence Zone (SPCZ) and trade winds [24]. Dispersal simulations carried out during this study were confined to the following area: 15° to 22° S and 175° W to 187° E, which extends farther east than Fiji's Exclusive Economic Zone (EEZ) and territorial waters and encompasses the Kingdom of Tonga. Tonga was included here, as a previous study reported genetic connectivity between Fijian and Tongan populations of *P*. *margaritifera* [6], supported by particle dispersal simulations.

### Model design and simulation approach

To evaluate larval transport pathways across Fiji and identify potential settlement locations, larval dispersal was simulated using the particle dispersal modelling software DisperGPU developed by Cyprien Bosserelle (https://github.com/CyprienBosserelle/DisperGPU), using the approaches described by Lal et al. [12] and Lal et al. [6], with a few modifications. These modifications are described under the relevant model component sections below. In summary, the approach involved two models: the DisperGPU particle dispersal model, and the HYbrid Coordinate Ocean Model (HYCOM) hindcast data, which provided hydrodynamic forcing to drive the former. Larvae were simulated as discrete particles, which were seeded into confined particle source locations (natal reefs) at the beginning of the simulations. Particle movements were subsequently tracked for a fixed period of time, to approximate the PLD of *P*. *margaritifera*. Numbers of particles visiting coordinates of interest were also counted, including the spat collector deployment sites studied by Kishore et al. [22], to permit comparison of actual vs. simulated ''potential recruitment".

### Hydrodynamic and particle dispersal numerical models

The DisperGPU particle dispersal model was driven by current velocity output from the global HYbrid Coordinate Ocean Model (HYCOM) data [25,26]. The HYCOM model had a resolution (*dx*) of 1/12th of a degree and output every day. The particle model used a standard Lagrangian formulation [11,27], where particle displacement is expressed as:

$$\Delta x=u_{p}*\Delta t+K$$ (1)

Here *x* represents particle position (latitude and longitude), Δ*x* is particle displacement during a time step Δ*t* (which was calculated so that Δ*x < dx*), and *u*_*p*_ is the surface current speed at the location of the particle. *K* is the eddy diffusivity which takes account of the random displacement of the particle due to turbulent eddies at a scale smaller than the hydrodynamic model resolution. *K* is calculated after Viikmäe et al. [28] as follows:

$$K=\sqrt{-4E_{h}\Delta t\log\left(1-R_{NA}\right)}\cos\left(2\pi R_{NB}\right)$$ (2)

Here, *E*_*h*_ is a horizontal turbulent diffusion coefficient, and *R*_*NA*_ with *R*_*NB*_ are uniformly distributed random numbers. The horizontal turbulent diffusion coefficient is unknown, but assumed to be 5 m^2^s^-1^ [28]; *u*_*p*_ is calculated by interpolating the velocity from the hydrodynamic model, both spatially and temporally. Gridded (10 Km^2^) surface currents from the HYCOM model were first interpolated to the dispersal step, after which the current velocity at each particle position was calculated using a bi-linear interpolation of the gridded surface currents, where only surface currents are taken into account and vertical movements neglected [14]. The particle age was retained and increased with simulation progression.

### *P*. *margaritifera* reproductive biology and model attributes

The black-lip pearl oyster is a broadcast spawner, with a PLD of 26–30 days [29,30]. This species is a protandrous hermaphrodite, and reproductively functional females are capable of producing upwards of 2.5–20 million eggs [31] in a single spawning event, depending on their age and size [32]. Developing larvae have very limited motility, and are largely dispersed by current advection and turbulent diffusion in the ocean surface (mixed) layer [6]. Spawning seasonality has also been documented in *P*. *margaritifera*, with two peak spawning periods observed in the Pacific Ocean [32]. In Fiji, these peaks occur from March to April, and November to December [6].

Considering these biological attributes, two separate dispersal simulations were run per year in March and November, respectively, to approximate the two peak spawning periods of *P*. *margaritifera* in Fiji. HYCOM data from four years (2012–2015) were utilised, to evaluate intra- and interannual variations in dispersal patterns. The years 2014–2015 also included an ENSO event [33,34], whereas 2012–2013 did not, permitting visualisation of potential ENSO-mediated changes in dispersal patterns. Furthermore, simulations utilising HYCOM datasets spanning late 2013 (spawning season 2) and 2014 (both spawning seasons), captured prevailing current patterns during the study carried out by Kishore et al. [22]. These authors deployed spat collectors between August and December 2013, with the gear soaked for 10–15 months [22]. Running dispersal simulations over this time period permitted a direct comparison of cumulative particle counts at spat collector deployment locations, with actual spatfall data.

### Particle dispersal model seeding, configuration and simulation runs

Simulations for each spawning season ran for 60 days, considering the PLD of *P*. *margaritifera* is 26–30 days. Particles were seeded everyday for the first 10 days, allowing a "settlement and recruitment" period of up to 30 days for the youngest particles seeded. No mortality or competency behaviour of the particles was simulated. The seed areas used for each simulation were identical, and seed area polygons were mapped from the shoreline to the 150 m depth contour (**S1 Fig**), using the Generic Mapping Tools (GMT) v.5.3.3 package [35]. Further precision in seeding the model was not possible, as the spawning dynamics and population densities of wild oysters in the Fiji Islands are unknown. This approach to seed area selection was adopted to account for both mapped and unmapped areas of coral reef habitat, assume a uniform distribution of spawning oysters in this habitat, and to extend seed areas into deeper water; as the HYCOM model is not adapted for shallow water environments and is therefore unable to resolve fine-scale hydrodynamic patterns <10 km [36].

A fixed number of 202,240 particles uniformly distributed across all seed area polygons was seeded each day, making a total of 2,022,400 particles released during simulations for each respective spawning season. This quantity of particles was selected based on constraints on available computational power, and DisperGPU input requirements (see https://github.com/CyprienBosserelle/DisperGPU for further information).

### Simulation post-processing and analyses

Particle positions were extracted daily from the beginning of each simulation, and plotted on a map of the study area using the GMT [35]. These plots were used to produce animations of each simulation run (see **S1–S8 Gifs**), and to visualise dispersal patterns between source and sink locations. Particle positions on the last day of each simulation (day 60) were extracted separately, and on these maps, the simulation animations were used to mark the location, orientation and trajectories of major particle flux patterns.

Cumulative particle counts (the numbers of particles visiting any particular 10 Km^2^ grid cell) were also recorded and plotted to visualise geographic regions of potential particle aggregation. Cumulative particle counts were plotted on heat maps at 5-day intervals, from day 30 to day 60 of each simulation using GMT v.5.5.3. Cumulative particle visit values on the last day of each simulation (day 60) were again extracted separately, and these heat maps annotated with spat collector deployment locations as per Kishore et al. [22]. This permitted visualisation of particle aggregation areas in the vicinity of the spat collection sites. Finally, for the three simulation datasets concurrent with spat collector deployment periods, cumulative particle counts were extracted for the collector deployment site coordinates for each respective simulation/spawning season. These were simulations for 2013 spawning season 2, and both seasons for 2014 to match the duration of collector gear soak times.

Box and whisker plots were generated for cumulative 60-day particle visit counts at each of the 28 collector sites, and superimposed with physical counts of recruited *P*. *margaritifera* spat. As Kishore et al. [22] carried out a single harvest at each collector site at the end of the 10–15 month soak period, actual recruitment data for each spawning season separately was not available. Recruitment of *P*. *margaritifera* spat in the Fiji Islands is stochastic, and therefore collectors often need to be deployed for extended period of up to 10 months, to ensure sufficient numbers of oysters of suitable size are collected [22]. Therefore, the same total spat counts were plotted against particle count data for all three simulated spawning seasons individually. To examine particle densities adjacent to spat collector deployment sites, cumulative particle visit counts were extracted for both "early" (day 30–40) and "late" (day 50–60) recruitment windows, and used to generate a pairwise particle density plot between pairwise collector sites. Median values were then calculated between site pairs across all three spawning seasons simulated over 2013–2014 and used to construct a pairwise matrix. This matrix was then plotted using the *gplots* and *RColorBrewer R* packages [37,38].

Pearson's product-moment correlations were used to test the relationship between cumulative particle counts, and the numbers of *P*. *margaritifera* spat recruited onto collectors across all deployment sites. A Wilcoxon rank sum test (with continuity correction) was also carried out on the three seasonal datasets combined. One sample t-tests were also carried out for each deployment site individually, to compare particle counts with numbers of spat collected. Data were tested for normality using the Shapiro-Wilk test, and for homogeneity of variances with Levene's test using the base *R* package for statistical computing [39]. Arcsinh transformations were used on spat recruitment data to correct deviations from normality.

## Results

### Dispersal flux patterns

**Fig 2:**
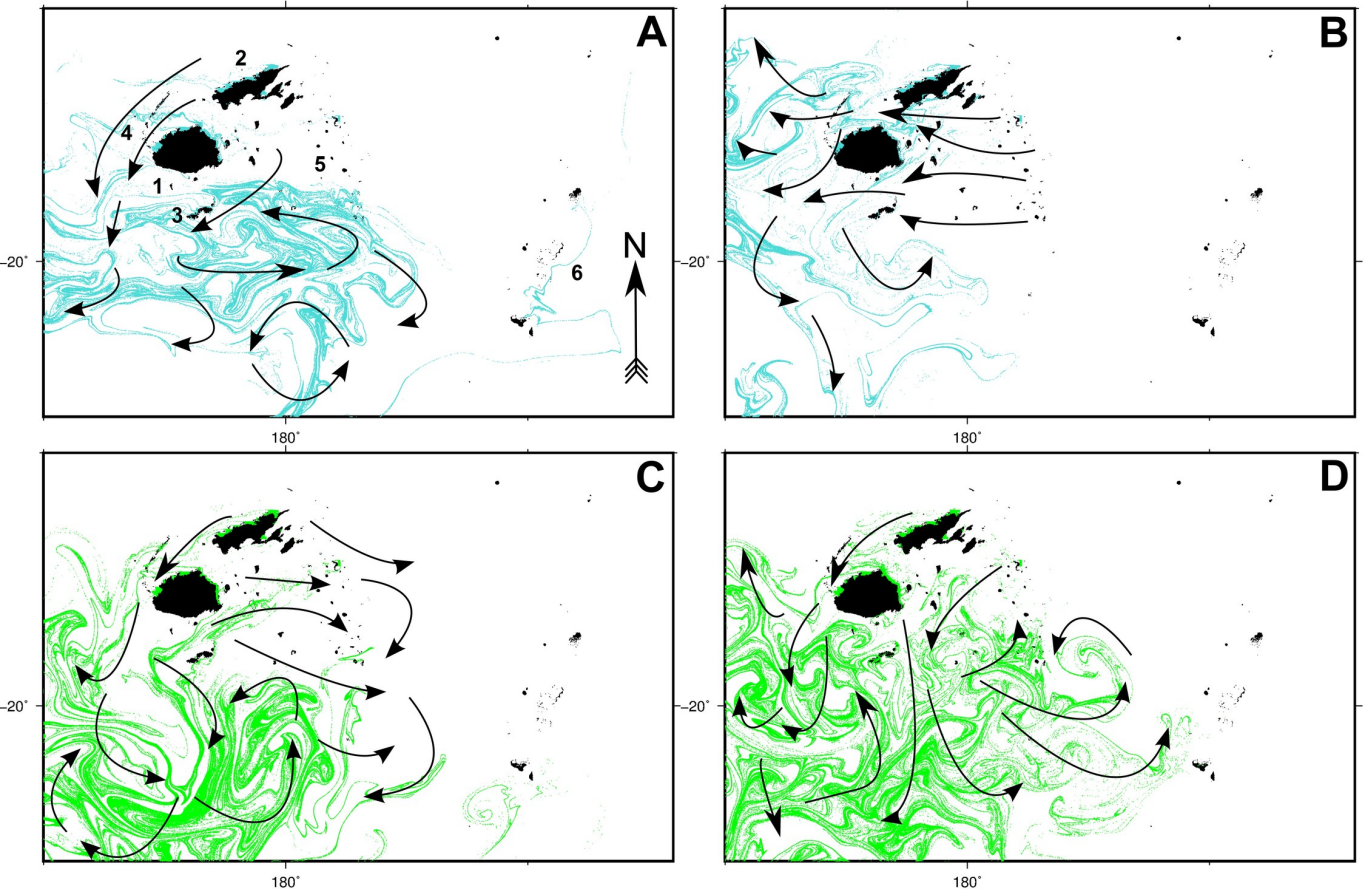
Particle dispersal simulation results for 2012 and 2013 datasets (non-ENSO years). Final (day 60) particle position plots are shown for spawning seasons 1 (**A**) and 2 (**B**) for 2012, and seasons 1 (**C**) and 2 (**D**) for 2013. All simulations were run for 60 days. Arrows denote the positions and trajectories of major particle flux patterns. Animations of these simulations are available as **S1**–S4 Gifs. Numbers denote the following localities: Viti Levu (1), Vanua Levu (2), Kadavu (3), Yasawa archipelago (4), Lau archipelago (5) and the Kingdom of Tonga (6).

**Fig 3:**
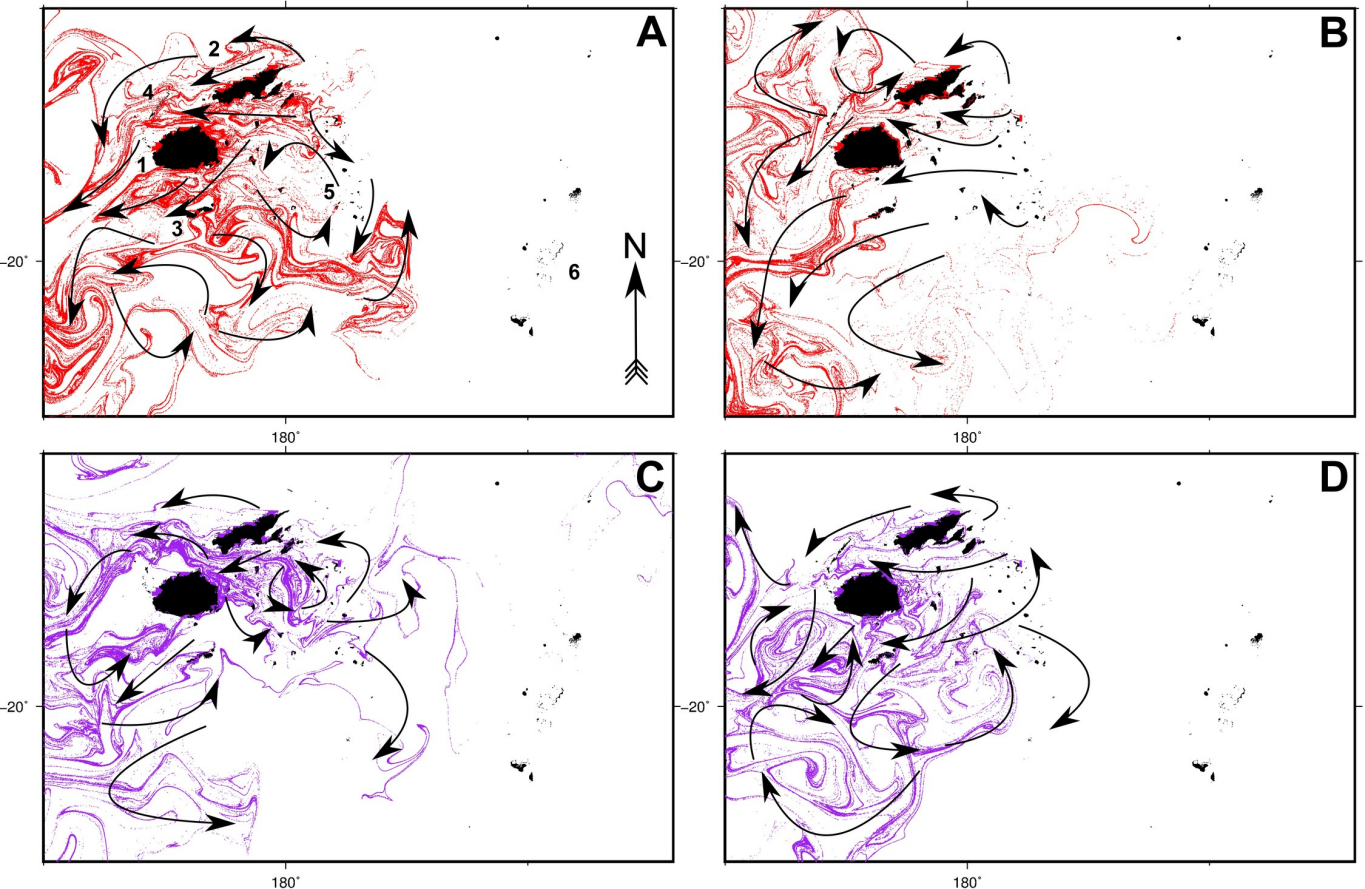
Particle dispersal simulation results for 2014 and 2015 datasets (ENSO years). Final (day 60) particle position plots are shown for spawning seasons 1 (**A**) and 2 (**B**) for 2014, and seasons 1 (**C**) and 2 (**D**) for 2015. All simulations were run for 60 days. Arrows denote the positions and trajectories of major particle flux patterns. Animations of these simulations are available as S5–S8 Gifs. Numbers denote the following localities: Viti Levu (1), Vanua Levu (2), Kadavu (3), Yasawa archipelago (4), Lau archipelago (5) and the Kingdom of Tonga (6).

Simulations of larval transport revealed a high degree of particle admixture by surface ocean currents across the Fiji Islands over all datasets (see **Figs 2** and **3**, and **S1–S8 Gifs** for animations). For all simulations, particle fluxes initially occurred primarily in a westward direction, followed by turbulent southwards flow, matching the trajectory of the east-to-west flowing South Equatorial Current (SEC). While this pattern was consistent across the majority of the eight simulations, differences were observed both intrannually between spawning seasons, and interannually between ENSO and non-ENSO event years.

Intrannual dispersal patterns displayed opposing trends. For example, data for the 2012 spawning season 1 showed particles dispersing initially southwards, with some easterly drift from day 45 onwards (**Fig 2A**, **S1 Gif**), whereas initial flows for season 2 tended westwards with minimal subsequent easterly drift (**Fig 2B**, **S2 Gif**). Similar differences were observed in the 2013 data, with the season 1 simulation demonstrating an east-south-west oscillation (**Fig 2C**, **S3 Gif**), while season 2 data indicated a primarily southerly and subsequent westward flux pattern (**Fig 2D**, **S4 Gif**). Particle movements during the 2014 and 2015 ENSO years were similarly different between spawning seasons. The season 1 simulation showed an east-west-south oscillation (**Fig 3A**, **S5 Gif**), whereas season 2 data depicted west-south-east-west movement (**Fig 3B**, S6 Gif). The 2015 season 1 dispersal pattern followed a south-east-west trend (**Fig 3C**, **S7 Gif**), while season 2 flows were directed initially west, and then south (**Fig 3D**, **S8 Gif**).

Differences in interannual dispersal patterns were primarily observed between non-ENSO (2012–2013) and ENSO (2014–2015) simulation years. Mass particle movements during non-ENSO years were in two directions, travelling either west or east initially, and then southwards (**Fig 2**, **S1–S4 Gifs**). Particle dispersal during ENSO years tended to oscillate, circulating in three or more directions as the simulations progressed, instead of travelling more directly away from landmasses into deeper water (**Fig 3**, **S5–S8 Gifs**). These patterns suggest the possibility of longer particle retention times in eastern Fiji during ENSO years, with the opposite occurring during non-ENSO years. Particle drift towards the Kingdom of Tonga also varied between ENSO and non-ENSO years. Of the four simulations carried out using 2012–2013 (ENSO year) data, all showed particles arriving in Tonga (in the vicinity of Tongatapu), except for the 2012 season 2 simulation. Conversely, only two simulations during ENSO years depicted particle arrival in Tonga. These included simulations for 2014 season 2, and 2015 season 1.

### Cumulative particle counts and aggregation areas

**Fig 4:**
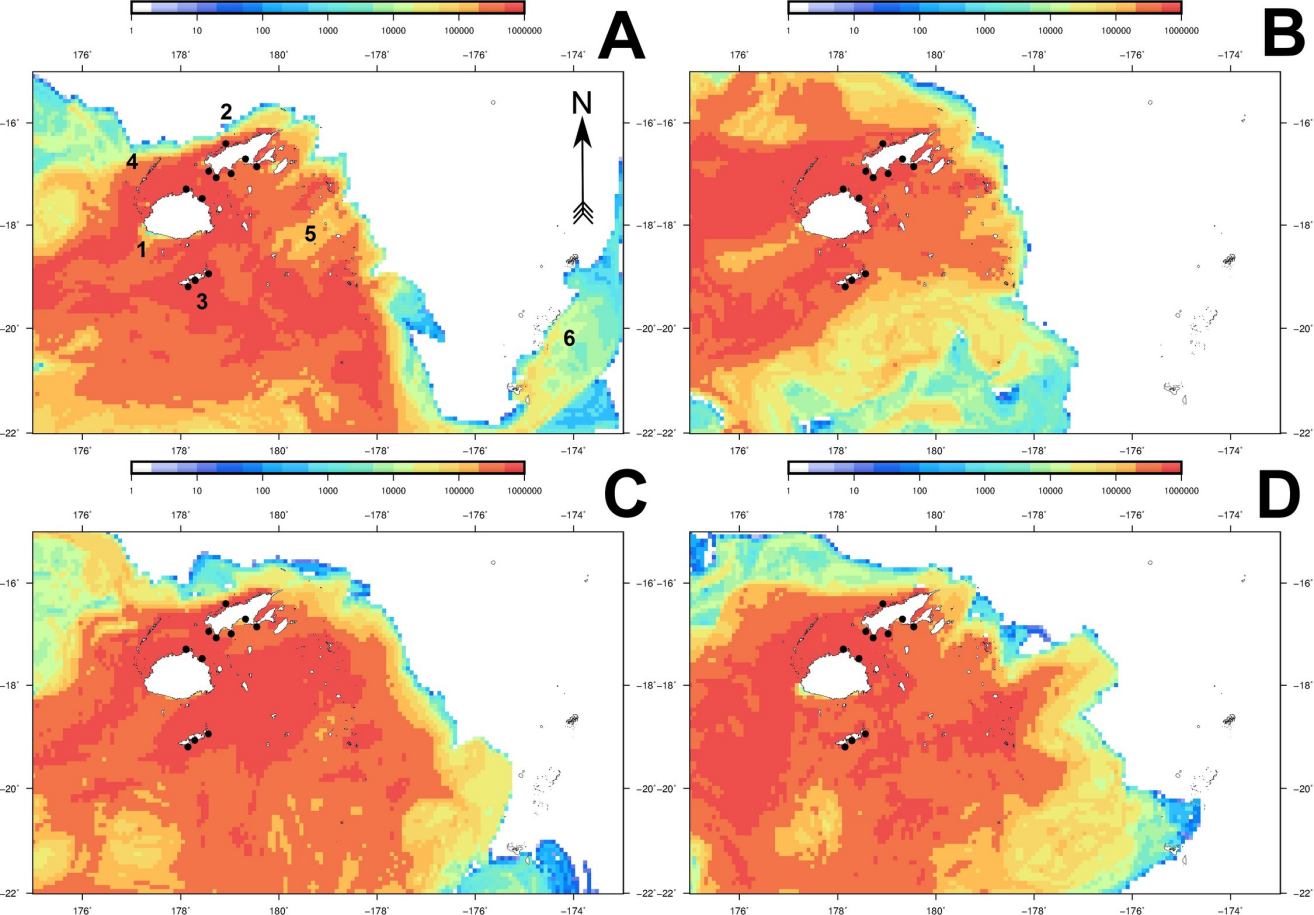
Cumulative particle count heat maps for 2012 and 2013 datasets (non-ENSO years). Final (day 60) particle counts are shown for spawning seasons 1 (**A**) and 2 (**B**) for 2012, and seasons 1 (**C**) and 2 (**D**) for 2013. Black circles denote the positions of spat collector deployments described by Kishore et al. [22]. The colour legend indicates the cumulative particle visit count within individual 10Km^2^ grid cells. Numbers denote the following localities: Viti Levu (1), Vanua Levu (2), Kadavu (3), Yasawa archipelago (4), Lau archipelago (5) and the Kingdom of Tonga (6).

**Fig 5:**
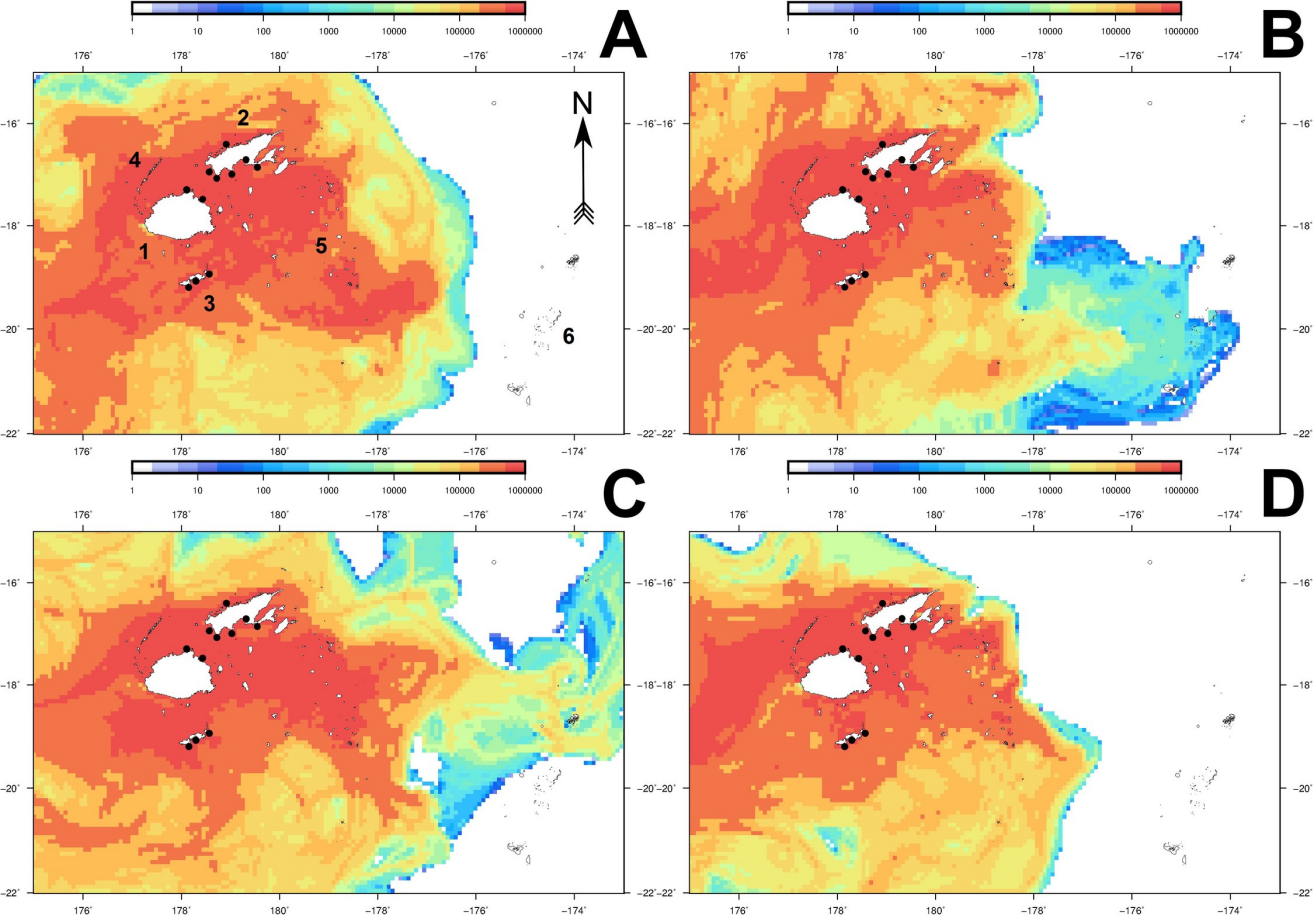
Cumulative particle count heat maps for 2014 and 2015 datasets (ENSO years). Final (day 60) particle counts are shown for spawning seasons 1 (**A**) and 2 (**B**) for 2014, and seasons 1 (**C**) and 2 (**D**) for 2015. Black circles denote the positions of spat collector deployments described by Kishore et al. [22]. The colour legend indicates the cumulative particle visit count within individual 10Km^2^ grid cells. Numbers denote the following localities: Viti Levu (1), Vanua Levu (2), Kadavu (3), Yasawa archipelago (4), Lau archipelago (5) and the Kingdom of Tonga (6).

The majority of particles released from all seed areas accumulated in shallow water along the coastlines of the major islands in the Fiji group, as well as south of Viti Levu. These observations were recorded during all simulations between days 30 to 60, with this window designated as the putative "recruitment" phase for each simulation. Cumulative particle counts in these aggregation areas were between 104 to 1,930% higher (**Figs 4** and **5**), compared to particle densities observed outside of these areas predominantly north of Vanua Levu and east towards Tonga.

For all simulations, between 15–31% of all particles released were retained in the Bligh Water channel located between Viti Levu and Vanua Levu by day 60, with particle densities approximating ≥1,000,000 cumulative particle visits/cell. Areas of high particle density (>560% compared to lower density areas) also extended westwards from the Bligh Water channel to the Yasawa archipelago, or east towards the Lau group of islands, depending on the prevailing flow pattern in effect for the spawning season and year (**Figs 4** and **5**). Regions where high numbers of particles aggregated (≥200% increase in particle numbers) matched the dispersal flux patterns captured by the simulation animations (**S1–S8 Gifs**).

Sites across Fiji which experienced consistently high numbers of particle visits (212.5–3,025% increases in visits/cell) demonstrated a consistent pattern between spawning seasons. During spawning season 1 simulations (**Figs 4A**, **4C**, **5A** and **5C**), a prevailing easterly surface current flow resulted in the Lau group of islands receiving high numbers of particle visits (>300,000 particle visit/cell) relative to other locations. Simulations for spawning season 2 showed a reversal of this trend, with a predominantly westward flow producing particle aggregations in the Yasawa archipelago (**Figs 4B**, **4D**, **5B** and **5D**). Patterns of particle aggregation in these areas, while not addressed during this study, are worthy of future finer-scale investigation for evaluation as additional spat collector deployment sites. In particular, the Yasawa archipelago, Gau in eastern Viti Levu and the northern Lau archipelago all demonstrated particle visit numbers >1,000,000/cell, and should be assessed (**Figs 4** and **5**).

### Comparison of dispersal simulation with actual spat recruitment at collector sites

**Fig 6:**
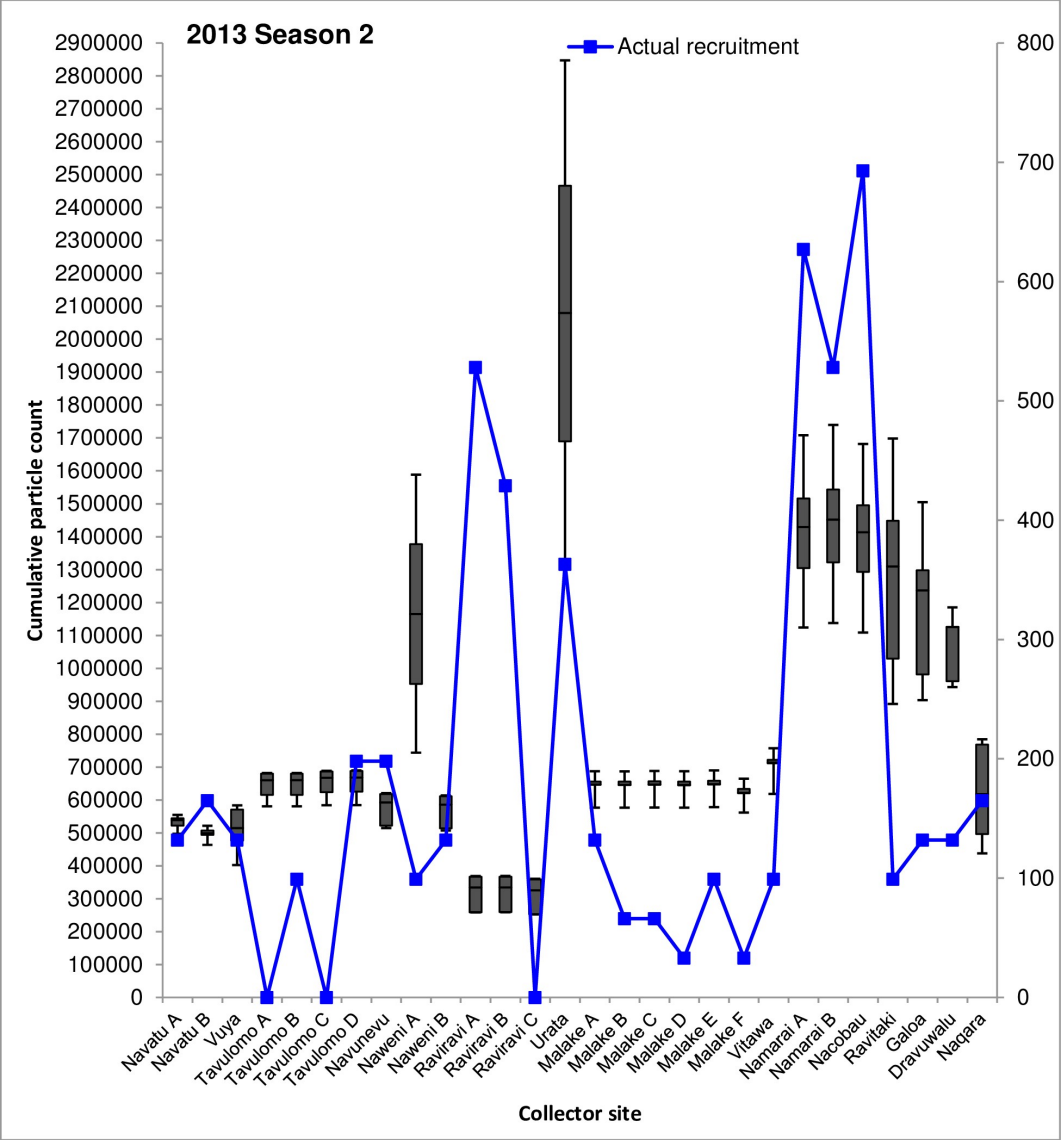
Simulated cumulative 60-day particle counts for the 2013 second seasonal spawning peak period (November-December) at 28 spat collector deployment sites. Spat collector sites are presented on the horizontal axis and the box and whisker plots for cumulative particle counts are displayed on the primary vertical (left) axis. Boxes indicate the limits of the first and third quartile values for cumulative particle counts at each collector deployment site, while upper and lower whiskers represent the maximum and minimum particle counts respectively. Physical counts of recruiting *P*. *margaritifera* spat are shown by the line plot in blue, and presented on the secondary vertical (right) axis for reference. Recruiting oyster counts are over a 10–15 month period from August 2013 to November 2014, and derived from Kishore et al. [22].

**Fig 7:**
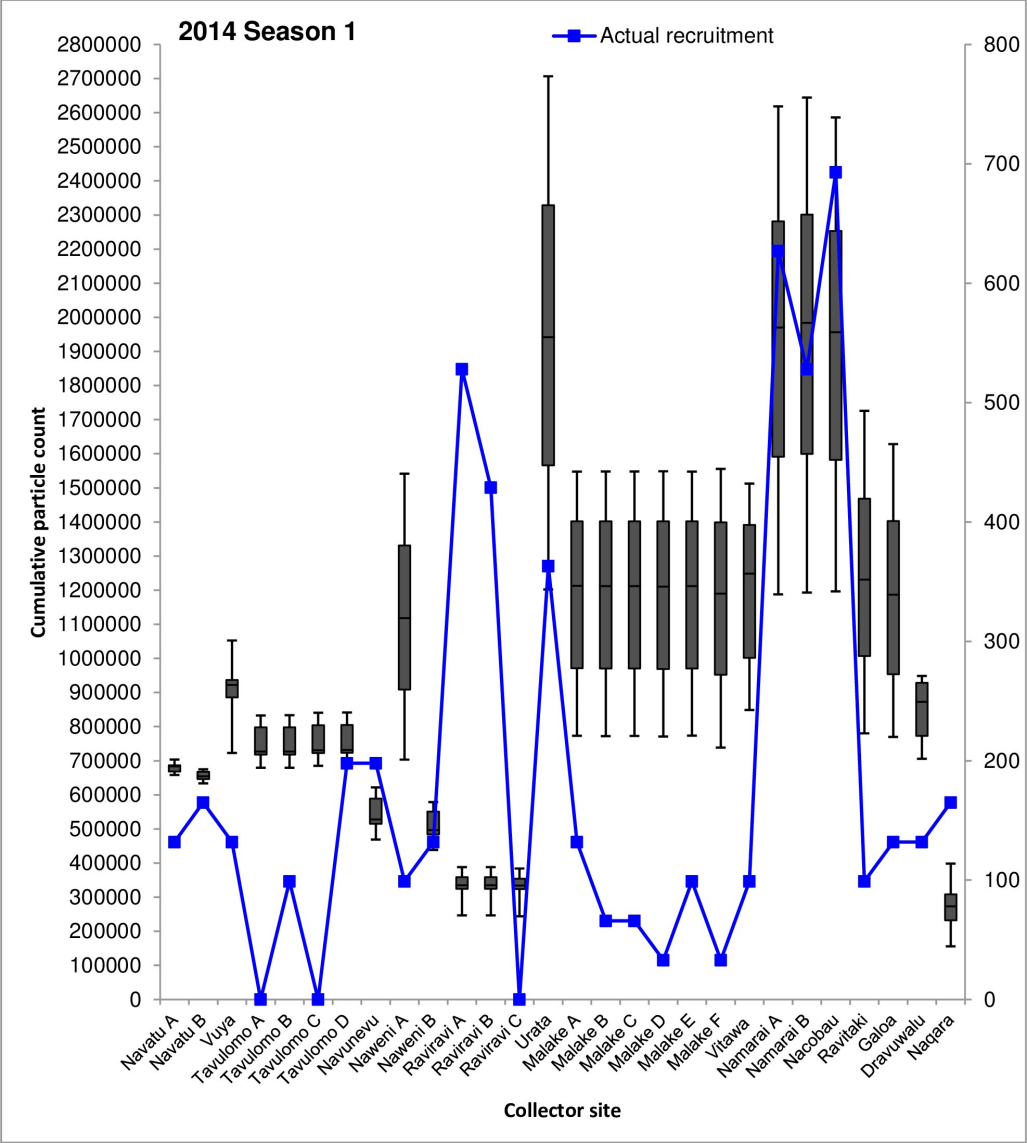
Simulated cumulative 60-day particle counts for the 2014 first seasonal spawning peak period (March-April) at 28 spat collector deployment sites. Spat collector sites are presented on the horizontal axis and the box and whisker plots for cumulative particle counts are displayed on the primary vertical (left) axis. Boxes indicate the limits of the first and third quartile values for cumulative particle counts at each collector deployment site, while upper and lower whiskers represent the maximum and minimum particle counts respectively. Physical counts of recruiting *P*. *margaritifera* spat are shown by the line plot in blue, and presented on the secondary vertical (right) axis for reference. Recruiting oyster counts are over a 10–15 month period from August 2013 to November 2014, and derived from Kishore et al. [22].

**Fig 8:**
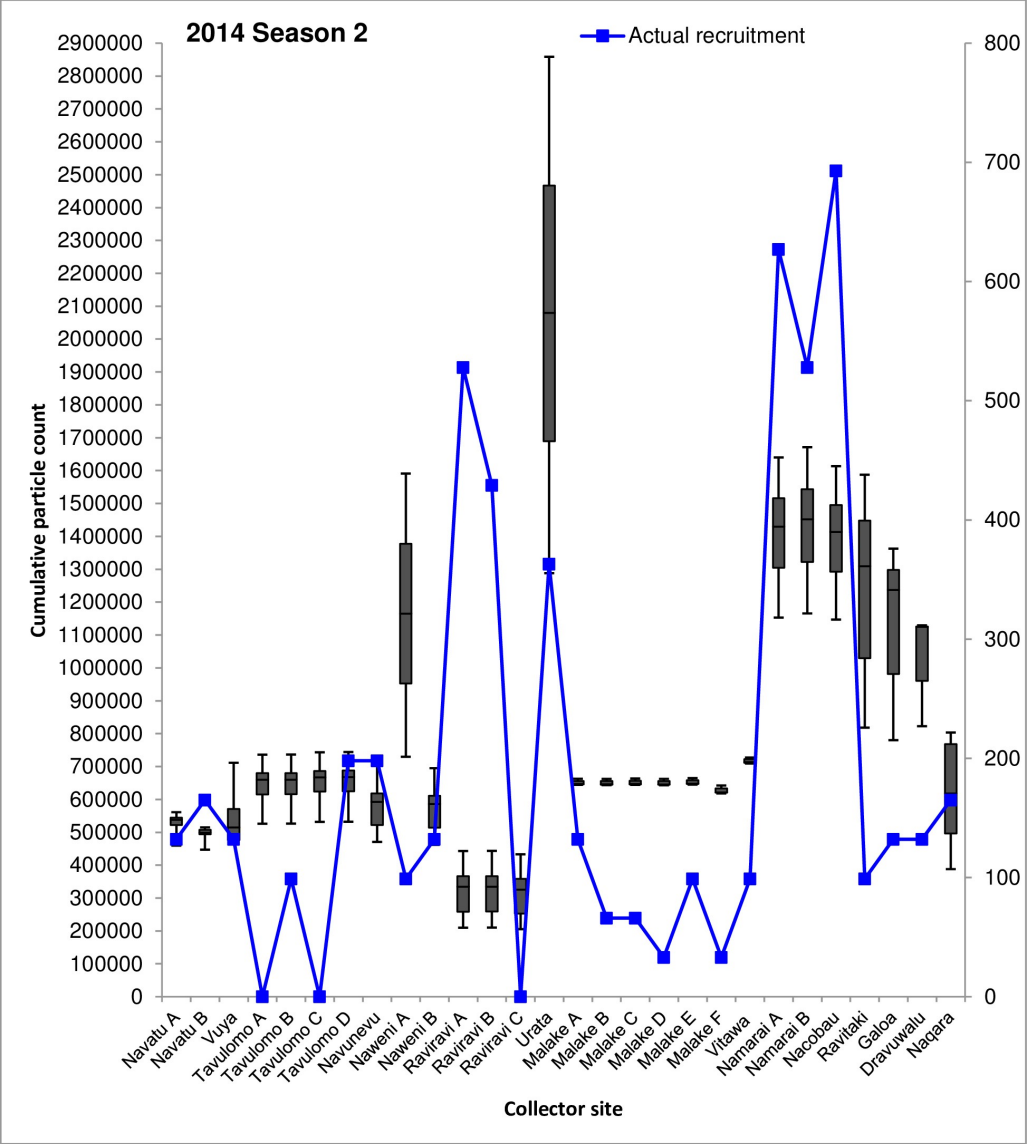
Simulated cumulative 60-day particle counts for the 2014 second seasonal spawning peak period (November-December) at 28 spat collector deployment sites. Spat collector sites are presented on the horizontal axis and the box and whisker plots for cumulative particle counts are displayed on the primary vertical (left) axis. Boxes indicate the limits of the first and third quartile values for cumulative particle counts at each collector deployment site, while upper and lower whiskers represent the maximum and minimum particle counts respectively. Physical counts of recruiting *P*. *margaritifera* spat are shown by the line plot in blue, and presented on the secondary vertical (right) axis for reference. Recruiting oyster counts are over a 10–15 month period from August 2013 to November 2014, and derived from Kishore et al. [22].

The majority of spat collector deployment sites examined by Kishore et al. [22] were positioned in locations which consistently received ≥109% increases in cumulative particle visits/cell across all simulations (**Figs 4** and **5**). Cumulative particle visit count data corresponding to spat collector deployment and soak times concordant with Kishore et al. [22] are presented in **Figs 6–8** for 2014 spawning seasons 1 and 2, and 2015 spawning season 2, respectively.

Median numbers of particle visits recorded on simulation day 60 between sites displayed remarkably similar patterns between all three spawning seasons. The highest median range of particle visits was consistently observed at Urata in Savusavu Bay, Vanua Levu (1,909,521–2,079,864); Namarai A, Namarai B and Nacobau in northern Viti Levu (1,351,352–1,983,574), together with Ravitaki, Galoa and Dravuwalu in Kadavu (454,823–1,309,570). All other sites recorded lower median numbers of particle visits across all three spawning season simulations, ranging from 241,947–1,248,810.

Seasonal trends were also evident in cumulative particle count data between sites. While some sites recorded relatively consistent median particle visit number ranges between spawning seasons, such as Naweni A (1,084,759–1,165,281), Urata (1,909,521–2,079,864), Namarai A (1,351,352–1,429,251), Namarai B (1,371,267–1,983,575) and Nacobau (1,361,911–1,956,238); others fluctuated. The largest variability over the study period was observed at collector sites in Malake A through F and Vitawa, which are all located near Ra on northern Viti Levu (**Figs 6–8**). Cumulative median particle visits among these sites during 2013 spawning season 2 and 2014 spawning season 2 were 772,186±66,477 and 646,935±28,449, respectively (**Figs 6** and **8**). During the 2014 spawning season 1 however, recorded particle visit numbers almost doubled to 1,212,008±17,360 (**Fig 7**). Similar variability, although to a lesser extent, was observed at Navatu A and B, Vuya, Tavulomo A-D, Navunevu and Naqara.

Simulated data presented here indicate that some sites may be more advantageous for spat collector deployment than others, due to consistently higher relative cumulative particle visit counts. Comparison of the simulated particle visit data to actual *P*. *margaritifera* spatfall recorded on collectors by Kishore et al. [22] across all three spawning seasons revealed several consistencies between collector deployment sites. At Namarai A, Namarai B and Nacobau (**Figs 6–8**), high particle counts (1,429,251±288,666) across all three spawning season simulations matched a similarly high mean number of spat collected among these three sites (n = 616). Conversely, at Navatu A, Navatu B, Vuya, Tavulomo A-D and Navunevu, lower particle counts (610,433±119,517) were concordant with lower average spatfall among these sites (n = 115).

Opposing trends were also observed, where relatively high particle visit counts did not match the relative numbers of spat harvested from collector gear. At Ravitaki, Galoa and Dravuwalu for example, 1,125,076±272,037 cumulative particle visits were recorded, approaching the values recorded for high-yielding sites at Namarai A, Namarai B and Nacobau, however 80% fewer spat were collected at these sites (n = 121) by Kishore et al. [22]. Examination of cumulative particle counts and numbers of *P*. *margaritifera* spat recruited onto collectors at each deployment site showed significant and positive correlations. For the second spawning peak simulation for the 2013 dataset, a moderately positive correlation was determined; *r*(26) = 0.435, p = 0.02, *R*^2^ = 0.189, explaining 18.9% of the variance in the data. Correlations were also moderately positive for both the 2014 datasets, with the second spawning peak simulation (*r*(26) = 0.438, p = 0.02, *R*^2^ = 0.1914) explaining similar proportions of variance to the first spawning peak simulation *r*(26) = 0.428, p = 0.02, *R*^2^ = 0.1831. A Wilcoxon rank sum test carried out on the three seasonal datasets combined showed that simulated particle and actual spat recruitment counts were significantly different between sites (W = 784, p < 0.00), as did one sample t-tests carried out within each site. However, some sites agreed better with simulation results than others. Collectors deployed at Navatu A, Vuya, Tavulomo D, Naweni B and Naqara demonstrated good concordance (**Figs 6–8**), whereas particularly poor agreement was observed at Tavulomo B, Naweni A, both Raviravi sites, all Malake sites, Vitawa, Nacobau and all Kadavu sites. These patterns were similar across all three spawning season simulations. Comparisons could not be made at three sites where zero spat were collected (Tavulomo A, C, and Raviravi C).

**Fig 9:**
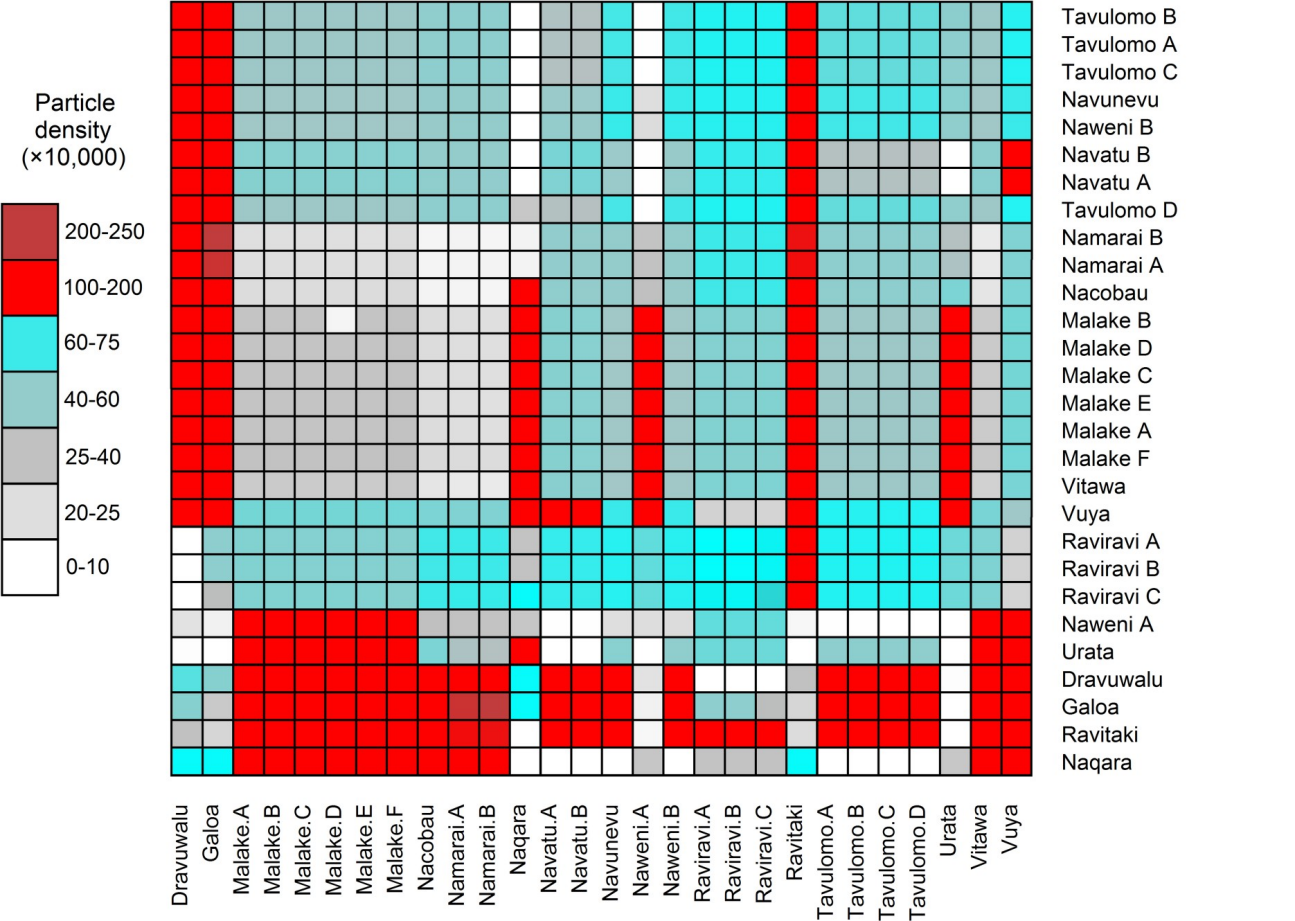
Pairwise matrix representation of modelled particle densities between 28 spat collector deployment sites during three spawning event simulations in 2013–2014. Cell colours correspond to particle counts recorded between days 30 through 60 for each spawning simulation between collector deployment site pairs.

Evaluation of median particle densities between pairs of collector deployment locations (**Fig 9**) provided additional information on putative larval retention patterns across Fiji. For example, substantially higher densities (100–200,000 particles/grid) were observed along the eastern coastline of Viti Levu, between Kadavu and Rakiraki (Malake vs. Ravitaki/Galoa/Dravuwalu sites), suggesting this region may be targeted for future trial spat collector deployments. Similarly, high particle densities (up to 200–250,000 particles/grid) were also recorded in the Bligh Water channel between Rakiraki and Tavulomo.

## Discussion

This study utilised hydrodynamic particle dispersal modelling to simulate the dispersal of black-lip pearl oyster larvae within the Fiji Islands over four years, and compared the results obtained with numbers of spat harvested from collectors during a previous study by Kishore et al. [22]. Model simulations identified putative current-driven larval dispersal and potential recruitment patterns, to inform the country's national spat collection programme through targeted collector gear deployments. Comparison of simulated data with physical spatfall across all collector sites reported by Kishore et al. [22] were weakly but positively correlated, indicating the utility of this model for optimising spat collection activity in Fiji. Recruitment at some sites however showed better agreement with simulated data than others. With further refinement and future finer-scale investigations, this dispersal model toolset may potentially be used in a predictive capacity, and be extended for application to other broadcast-spawning marine taxa with life history traits similar to *P*. *margaritifera*.

### *P*. *margaritifera* dispersal patterns and model utility

Particle dispersal simulations utilised here indicated a high degree of larval admixture over the Fiji Islands, primarily driven by prevailing surface ocean currents. These simulations agreed with earlier results reported by Lal et al. [12] and Lal et al. [6], which outline the presence of a single genetic stock of *P*. *margaritifera* across the Fiji Islands and Tonga, arising from widespread larval dispersal. Intra- and interannual differences in dispersal patterns revealed larval transport variability according to spawning season, along with influences from ENSO circulation. This information may be directly used to inform decision making on spat collector deployment locations and timing, to take advantage of seasonal larval drift patterns for maximising spat settlement on collection gear.

Thomas et al. [7] also used a hydrodynamic dispersal model to simulate larval connectivity for *P*. *margaritifera* in Ahe atoll, French Polynesia, and reported destination locations, spawning sites and PLD as the primary drivers of potential larval connectivity. Spatial patterns of larval connectivity described by these authors however, were different to the dispersal patterns reported here, likely due to study area bathymetry and geomorphological differences between a relatively ''closed'' atoll, and "open" volcanic island reef systems such as those present in Fiji. At Ahe atoll, the lagoon's primary water circulation is wind-driven, and simulations showed that larvae aggregated along unidirectional gradients constrained within the lagoon [7,40]. Given the South Equatorial Current's influence across Fiji [41] and free circulation between barrier and fringing reefs, larval transport during this study occurred in several directions. This difference between ''open'' and ''closed'' reef systems is known to play an important role in the recruitment success of spat onto collector gear, with higher recruitment rates observed in closed atoll systems compared to open reefs [42–45]. Despite this inherent limitation, Friedman and Southgate [42] suggest that with careful site selection and timing of spat collection gear deployment, commercial quantities of *P*. *margaritifera* spat may be harvested for culture. Biophysical modelling tools such as the dispersal model described here can assist with making determinations on optimal site selection and gear deployment timing, to maximise spat harvests.

### Future developments

Biophysical models are invaluable for providing insights into larval connectivity and recruitment in other marine taxa, to inform fishery management and culture practices. Hydrodynamic dispersal models have successfully been used to understand shrinking populations of the fluted giant clam *Tridacna squamosa* in Singapore [10], invasion patterns of the alien slipper limpet *Crepidula fornicata* in Europe [46], dispersal dynamics of coral larvae in Australia [14] and the Caribbean, respectively [47], and to inform restocking efforts for the cockle *Austrovenus stutchburyi* in New Zealand [19]. Other studies have used dispersal models to investigate age-specific dispersal patterns in the reef emperor fish *Lethrinus nebulosus* [48], and to evaluate the design of harvest refugia for large abalone species in Japan [49].

While the dispersal model used during this study has provided unique insights into the potential larval dispersal patterns of *P*. *margaritifera* in the Fiji Islands, there remains substantial room for refinement of the model and simulation parameters. An important area for improvement is the inclusion of larval behaviour in the particle model, such as particle mortality, swimming behaviour, particle homing ability when within fixed distance to a nearby reef towards the end of the dispersal phase, and settlement condition inputs such as reef depth or food availability. The addition of larval behaviour parameters will permit more accurate simulations of larval dispersal and recruitment, and enable higher precision in identification of suitable spat collector deployment sites.

Previous studies have incorporated larval behaviour into dispersal models with varying degrees of success. In their study of *P*. *margaritifera* larval connectivity in French Polynesia, Thomas et al. [7] discovered that vertical diel migration in their simulations did not affect connectivity patterns. However, these authors stated that several factors including light, food, salinity discontinuities, water temperature, predators and larval size/stage may all play a role in vertical larval migration behaviour, and potentially impact dispersal outcomes. Thomas et al. [7] reported that while there is currently no evidence that *P*. *margaritifera* larvae change their behaviour with larval size or developmental stage, at their study site, the highest physical spat recruitment rates were observed at a depth of 5 m, and tapered off at greater depths. Given this observation, results presented here suggest that simulations utilising only near surface current patterns, rather than a full 3D model seeded with uniformly distributed larvae at depth, may adequately capture larval transport fluxes in Fiji. Depth is known to be a major influence on larval pearl oyster settlement [50]. Tomaru et al. [51] studied Akoya pearl oyster (*P*. *fucata martensii*) spat-fall between depths of 1–30 m, and found the highest recruitment rates on collectors at 6 m, with optimal densities in the 1–10 m range; while significantly fewer spat were recorded at 15, 20 and 30 m. If depth-related competency information can be incorporated in the development of dispersal models for *P*. *margaritifera*, it may permit more realistic simulations of larval recruitment.

A more comprehensive biophysical model for *P*. *margaritifera* incorporating behaviour was developed by Thomas et al. [9], following their earlier study [7]. It incorporated a vertical swimming sub-model and a bioenergetics model to simulate larval feeding and growth. The bioenergetics model discriminated between feeding and non-feeding larval stages, and was forced using *in vivo* chlorophyll-a measurements as food concentration inputs and *in situ* water temperature data. These authors then applied a population dynamics model during simulation post-processing, to understand larval supply, mortality and settlement. Application of this model identified that a fortyfold increase in spat recruitment was realised during a food-abundant period, compared to a less abundant interval [9]. Neo et al. [10] in their investigation on giant clam recruitment in Singapore discovered strong interactions between spawning times, local geomorphology and poor fertilisation success on larval settlement rates. Through incorporation of spawning seasonality, vertical larval migration, larval growth and mortality parameters as larval competency inputs, they predicted that natural recovery of local giant clam stocks is unlikely. Development of such multilayered models using several forcing inputs (e.g. chlorophyll concentrations as food availability proxies, depth and water temperature), holds great promise for accurately modelling marine larval dispersal and recruitment rates for aquaculture and fisheries management applications.

Development of a more comprehensive larval dispersal model for Fiji is unfortunately hindered by the availability of a fine-scale hydrodynamic model, which would permit more detailed investigation of dispersal and recruitment patterns at the scale of individual reefs. The global HYCOM model used here is currently the only hydrodynamic model available for Fiji, is limited by a grid/cell size of 10 Km^2^ and not adapted for use in shallow water environments [26,36]. For comparison, Thomas et al. [7] and Thomas et al. [9], together with Neo et al. [10] used the MARS3D [40,52], and Delft3D-FLOW hydrodynamic models, respectively, both of which have a horizontal grid size of 100 m^2^. The development of fine-scale hydrodynamic regional ocean circulation models is a complex and expensive task, requiring considerable oceanographic resources and expertise. Baseline data on bathymetry, geomorphology, wave regimes, currents and numerous other inputs are required to begin model construction [7,53], which in many cases may be prohibitive due to cost and available technical capacity for developing small Pacific island states such as Fiji. Given the versatility of a fine-scale hydrodynamic model for a wide range of research and management applications apart from aquaculture, such as evaluating pollution dynamics, sea level rise, tidal inundation and weather prediction [53,54], perhaps a collaborative approach between several governmental and non-governmental agencies may provide a solution.

Once a higher resolution hydrodynamic model is available, an additional future direction for enhancement of the DisperGPU model is the development of predictive capacity for forecasting larval dispersal and recruitment patterns. Bidegain et al. [55] utilised a combination of larval behaviour, mortality and recruitment-settlement sub-models in the LARVAHS particle tracking model, to produce estimates of seasonal recruitment densities in two species of *Ruditapes* clams. Arnold et al. [56] similarly applied a biophysical model incorporating larval growth, swimming ability, food availability, water temperature and salinity inputs, to predict optimal reef restoration sites for the eastern oyster *Crassostrea viriginica* in the Gulf of Mexico. Using a different spatio-temporal Bayesian model and oceanographic data, Atalah et al. [57] forecasted biofouling blue mussel (*Mytilus galloprovincialis*) recruitment on green-lipped mussel (*Perna canaliculus*) farms in New Zealand. Once available, an enhanced larval dispersal and recruitment model, apart from being incorporated into the Fijian national spat collection programme for targeted collector deployments, could also be used as a fishery management tool. In this respect, identification of key source and sink reefs could be used to determine conservation areas for wild *P*. *margartifera* broodstock, and to determine priority areas for restocking or replenishment of depleted populations.

## Conclusions

This study presents a preliminary investigation of *P*. *margaritifera* larval dispersal and recruitment in the Fiji Islands using a simple hydrodynamic particle dispersal model, to inform spat collection efforts for the country's cultured pearl industry. Simulations described country-wide patterns of potential larval dispersal and settlement, which can directly inform the national spat collection programme. Comparison of simulated and physical spatfall at 28 spat collector deployment sites showed positive agreement between the two datasets, indicating future utility of the model for informing aquaculture and fishery management guidelines for the Fijian *P*. *margaritifera* resource.

Beyond the current study, there is substantial scope for enhancement of the model through incorporation of larval behavioural information, use of a finer-scale hydrodynamic model once available, and extension to other broadcast spawning taxa of importance for fisheries and aquaculture. While use of the global HYCOM model limited the resolution of finer-scale potential dispersal and recruitment patterns during this study, the model in its current form may be applied to other regions where more precise oceanic circulation models remain unavailable. Until high-resolution regional circulation models become available for these locations, the DisperGPU model provides a versatile and highly informative toolset for understanding the complexities of larval dispersal and settlement in fisheries and aquaculture applications.

## Supporting information

S1 Fig Particle dispersal simulation study area bathymetric chart and hydrodynamic model seed area polygons (inset). On the chart image, reef areas are highlighted in black, with shallow water depth contours <150m (500ft) highlighted in blue. Depth contours are presented in feet. Shallow water particle seed polygons are presented in blue on the inset, to capture the largest possible extent of suitable *P*. *margaritifera* reef-associated habitat. The chart image is adapted from area chart NZ 14638 Fiji to Kermadec Islands including Tongtapu at 1:1,500,000 scale, and is based upon official Paper Navigational Charts published by the New Zealand Hydrographic Authority at Land Information New Zealand (LINZ). It contains data sourced from LINZ under CC-By, and available online at https://data.linz.govt.nz/layer/51355-chart-nz-14638-fiji-to-kermadec-islands-including-tongatapuParticle dispersal simulation files. Please note that these.GIF files need to be opened in a web browser to display correctly.
(JPG)

S1 File Link to Kishore *et al*. (2018). (DOCX)

S1 Gif 2012 spawning season 1 simulation. (GIF)

S2 Gif 2012 spawning season 2 simulation. (GIF)

S3 Gif 2013 spawning season 1 simulation. (GIF)

S4 Gif 2013 spawning season 2 simulation. (GIF)

S5 Gif 2014 spawning season 1 simulation. (GIF)

S6 Gif 2014 spawning season 2 simulation. (GIF)

S7 Gif 2015 spawning season 1 simulation. (GIF)

S8 Gif 2015 spawning season 2 simulation. (GIF)
