# Supplementary figures and images for: Understanding marine larval dispersal in a broadcast-spawning invertebrate: A dispersal modelling approach for optimising spat collection of the Fijian black-lip pearl oyster Pinctada margaritifera
Source: PLoS One. 2020 Jun 18;15(6):e0234605. doi: 10.1371/journal.pone.0234605 (PMC7302709; doi:10.1371/journal.pone.0234605)

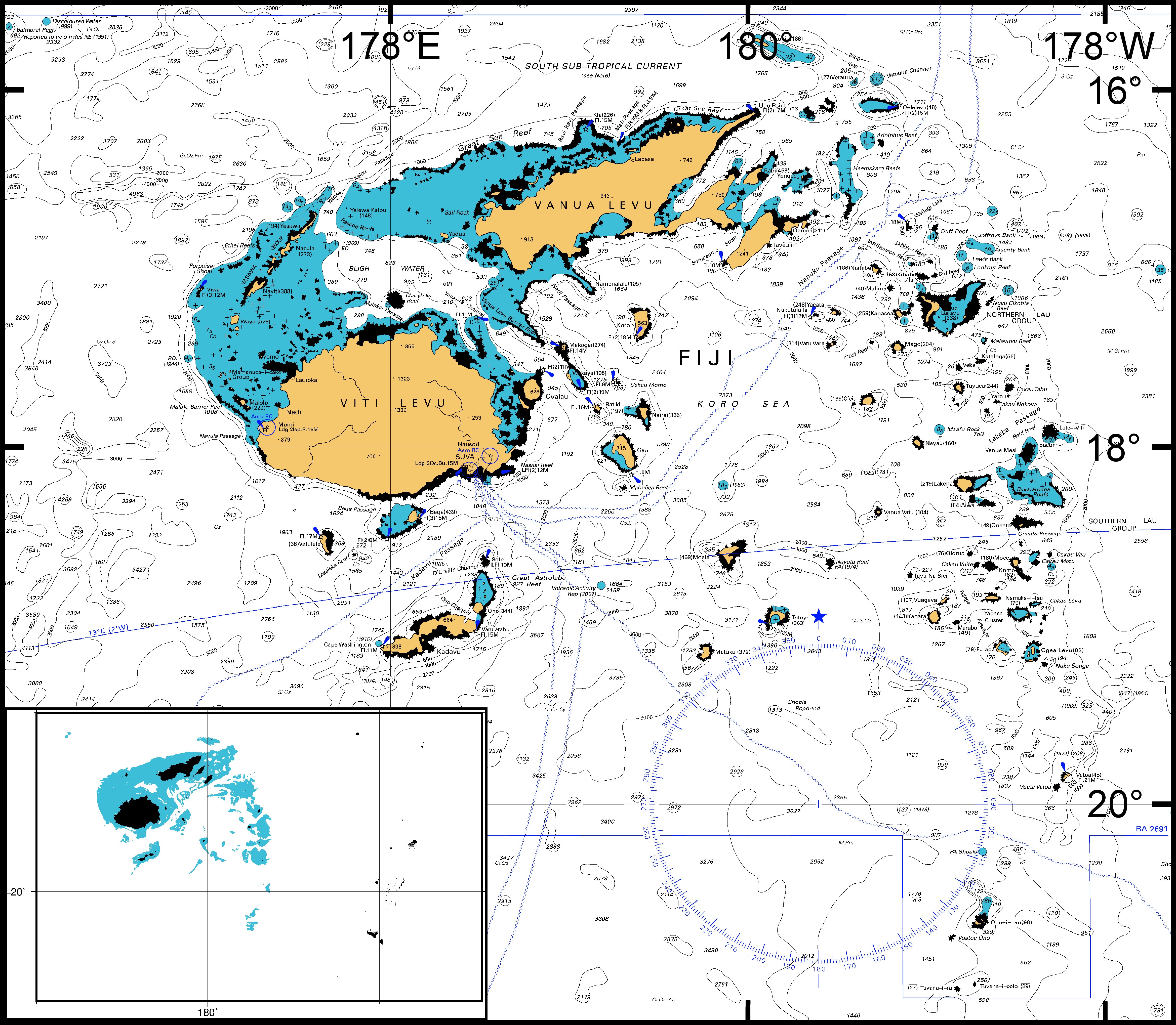

Supplement: S1 Fig — On the chart image, reef areas are highlighted in black, with shallow water depth contours <150m (500ft) highlighted in blue. Depth contours are presented in feet. Shallow water particle seed polygons are presented in blue on the inset, to capture the largest possible extent of suitable P. margaritifera reef-associated habitat. The chart image is adapted from area chart NZ 14638 Fiji to Kermadec Islands including Tongtapu at 1:1,500,000 scale, and is based upon official Paper Navigational Charts published by the New Zealand Hydrographic Authority at Land Information New Zealand (LINZ). It contains data sourced from LINZ under CC-By, and available online at https://data.linz.govt.nz/layer/51355-chart-nz-14638-fiji-to-kermadec-islands-including-tongatapuParticle dispersal simulation files. Please note that these.GIF files need to be opened in a web browser to display correctly. (JPG) [file pone.0234605.s001.jpg]

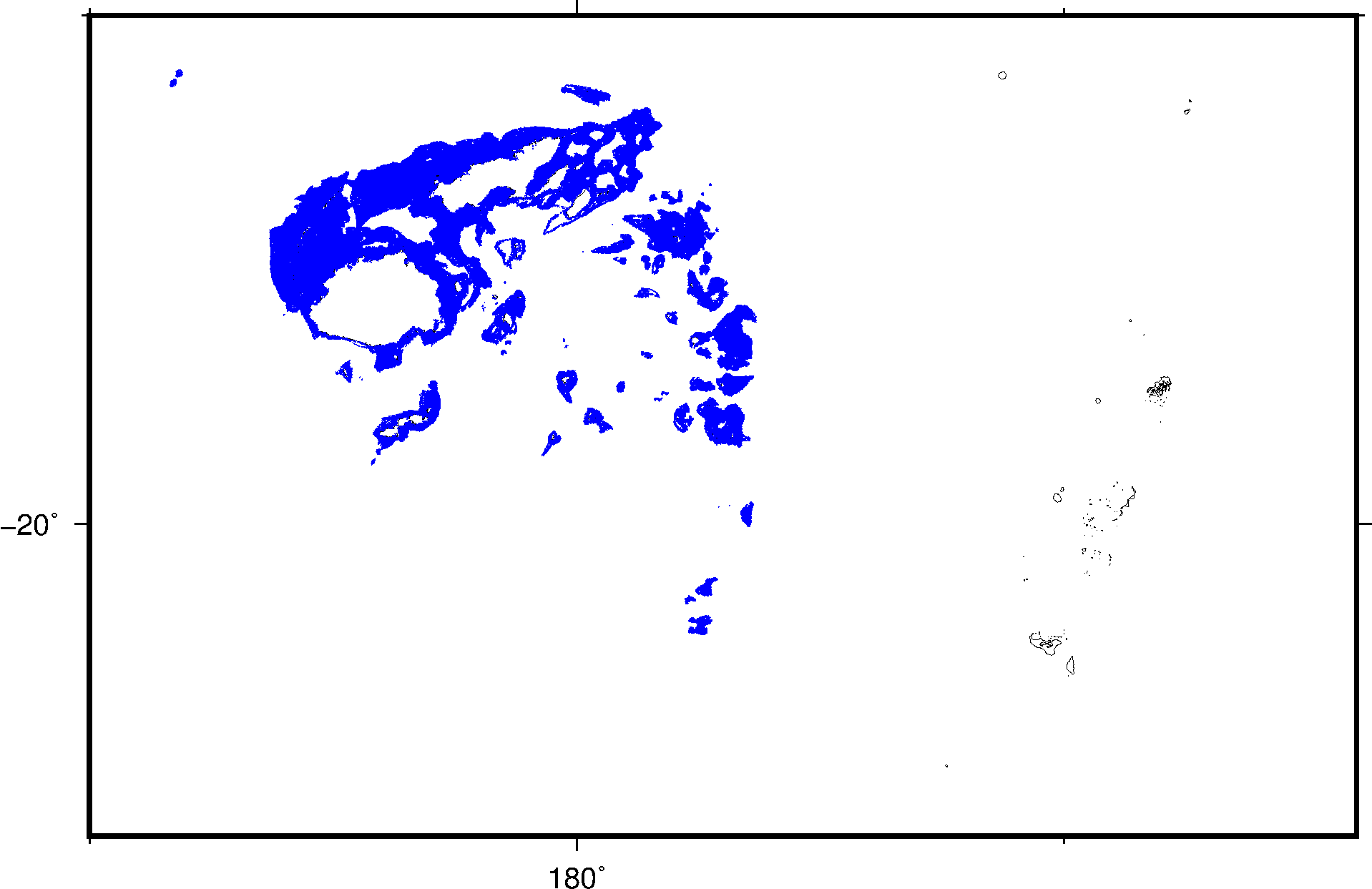

Supplement: S1 Gif — (GIF) [file pone.0234605.s003.gif]

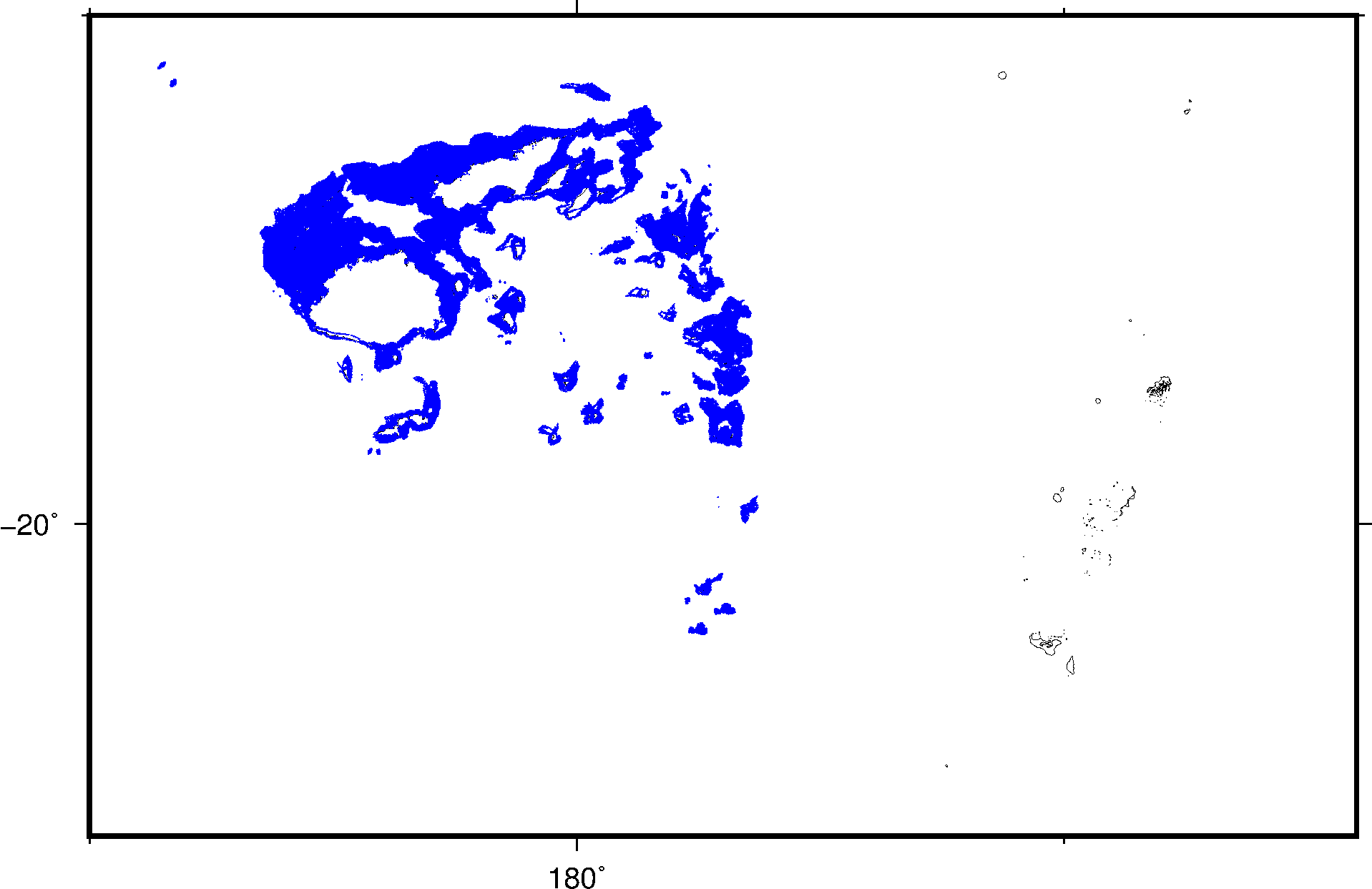

Supplement: S2 Gif — (GIF) [file pone.0234605.s004.gif]

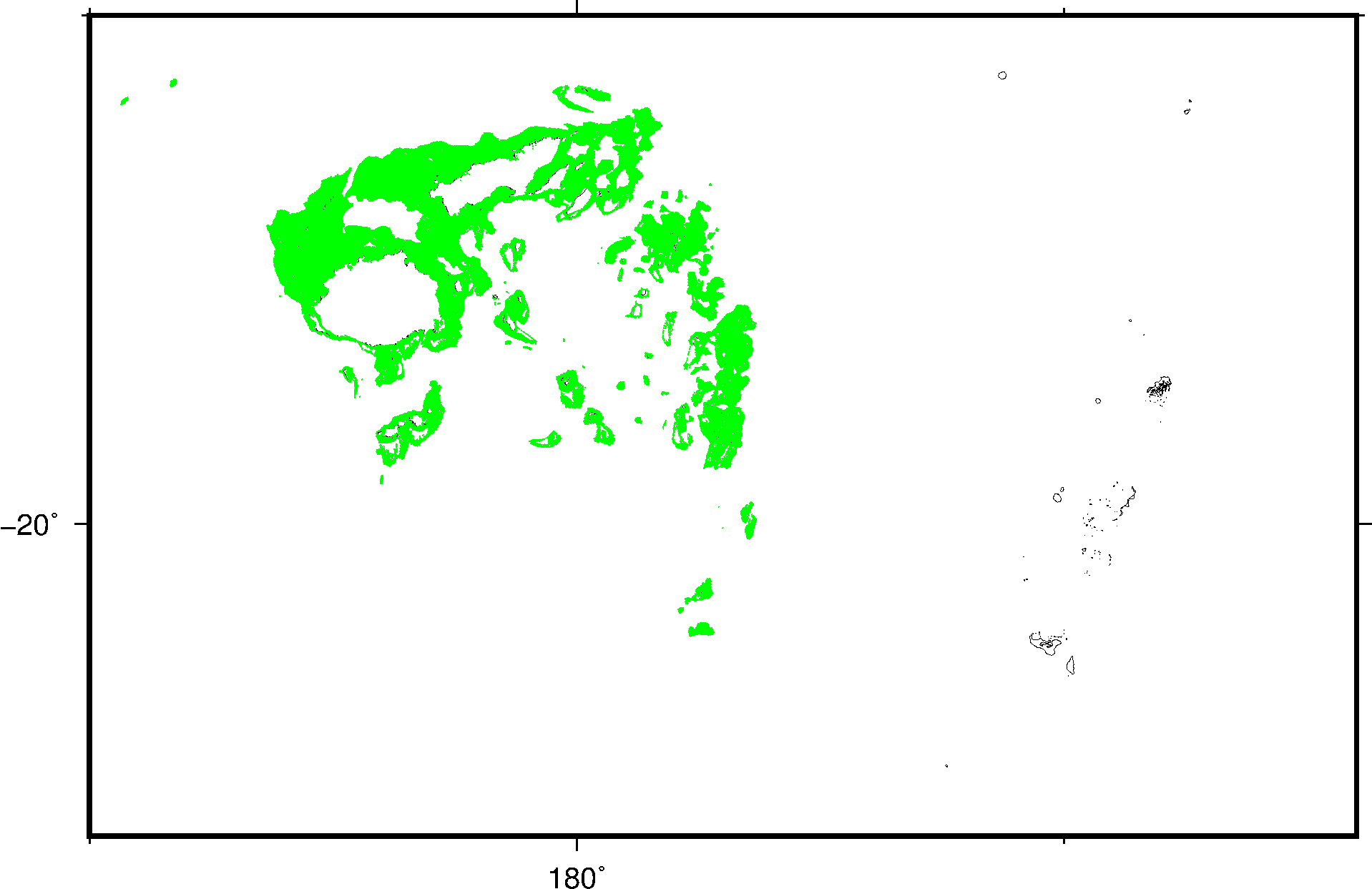

Supplement: S3 Gif — (GIF) [file pone.0234605.s005.gif]

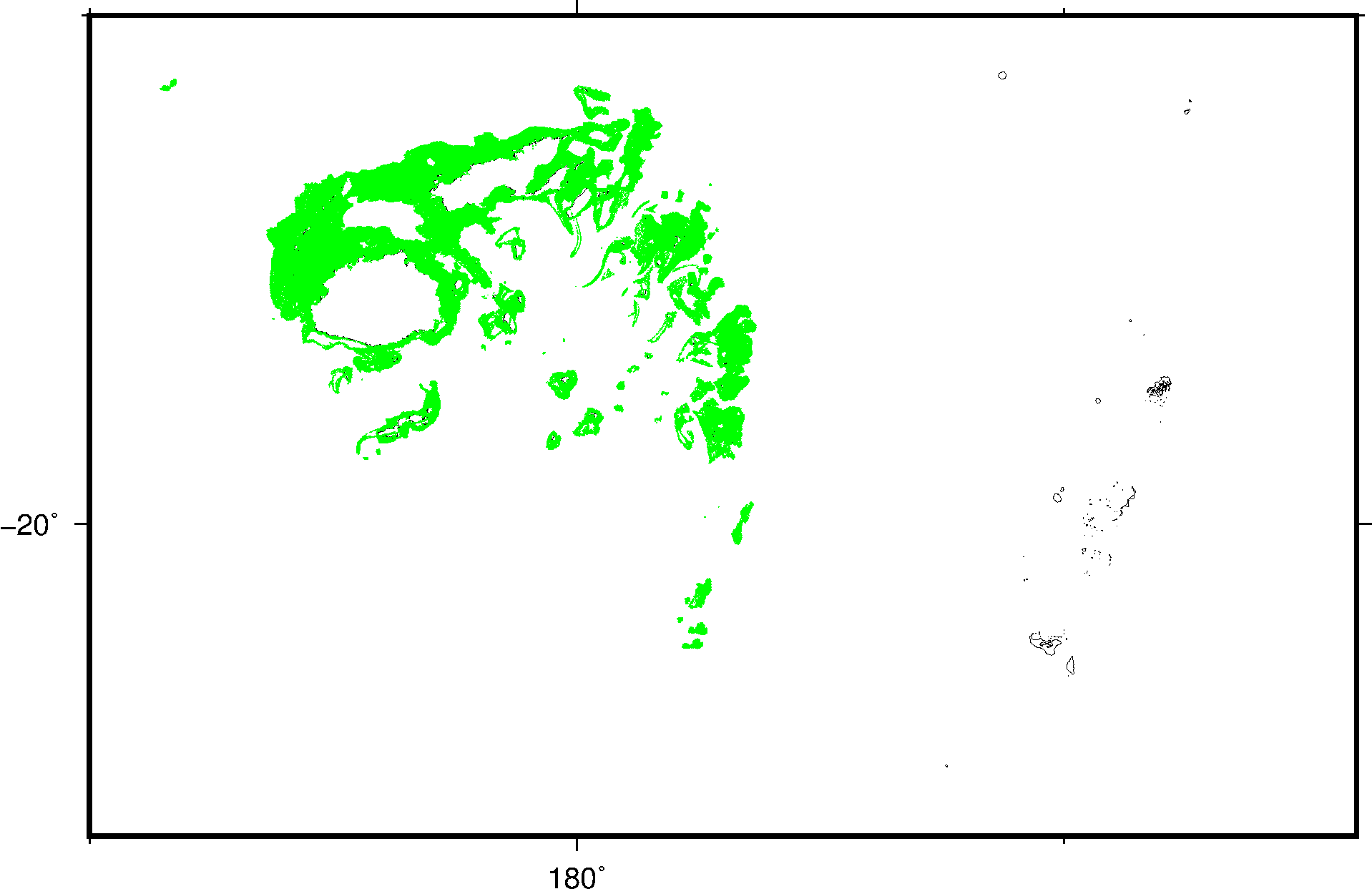

Supplement: S4 Gif — (GIF) [file pone.0234605.s006.gif]

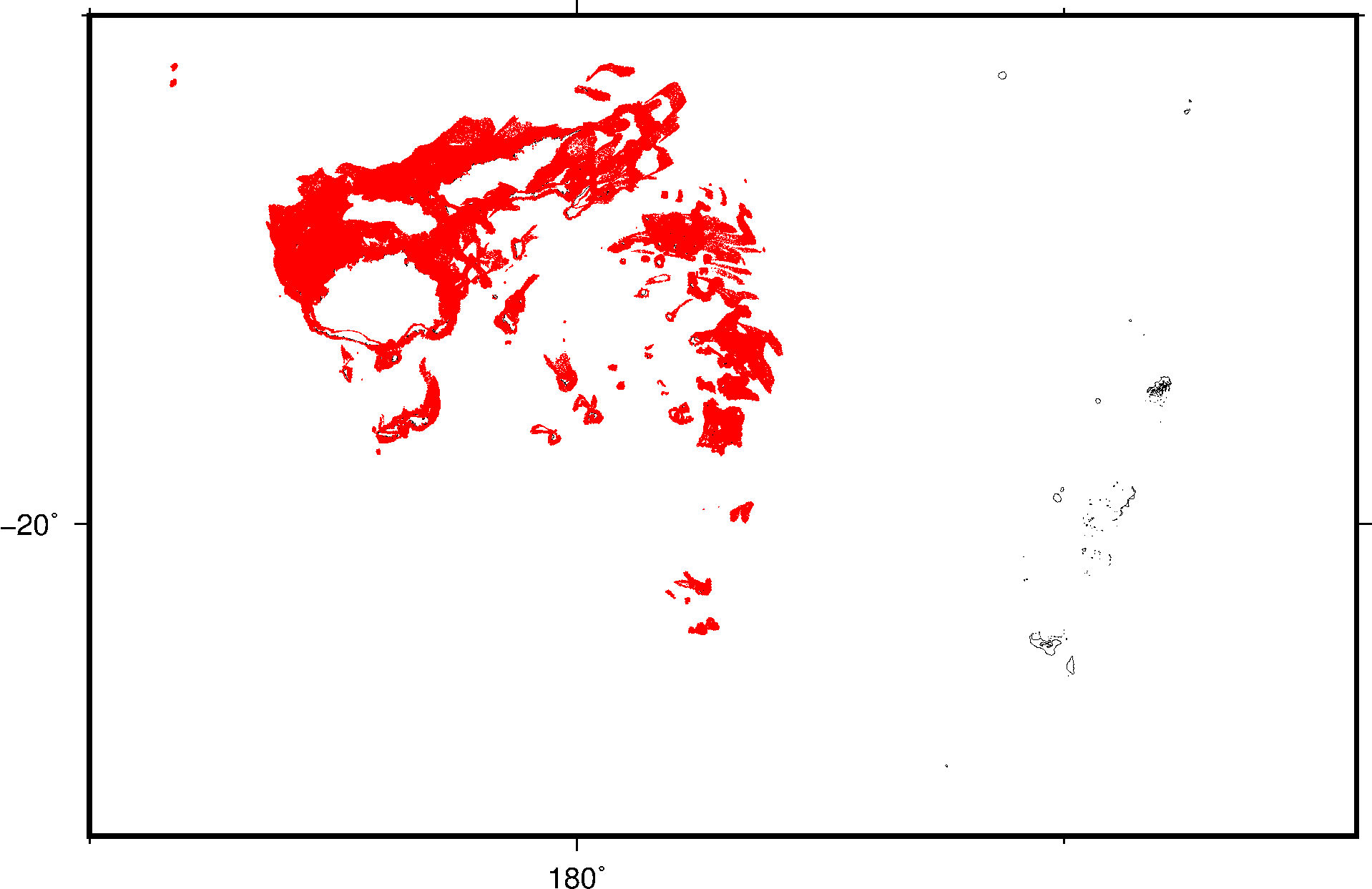

Supplement: S5 Gif — (GIF) [file pone.0234605.s007.gif]

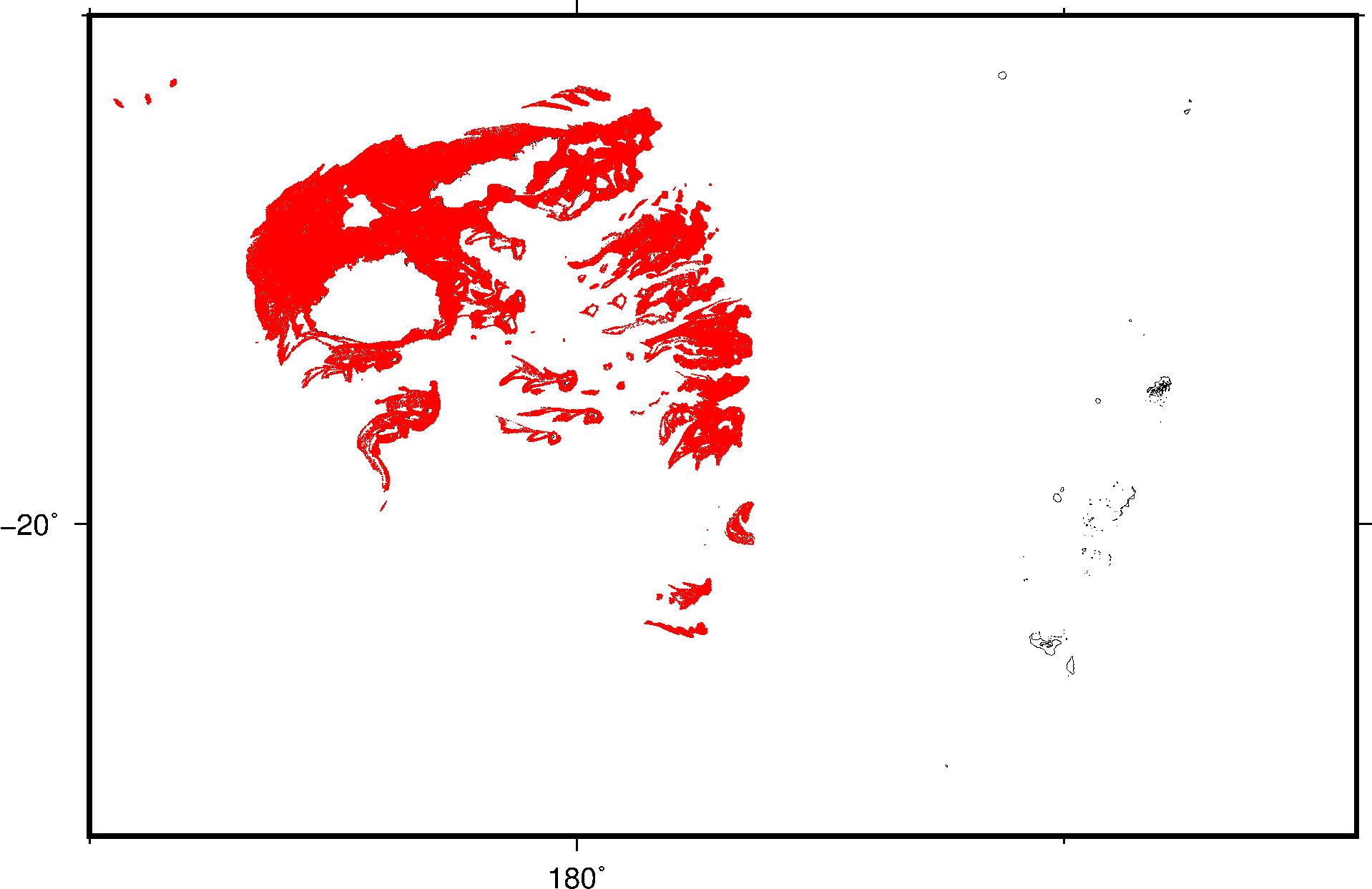

Supplement: S6 Gif — (GIF) [file pone.0234605.s008.gif]

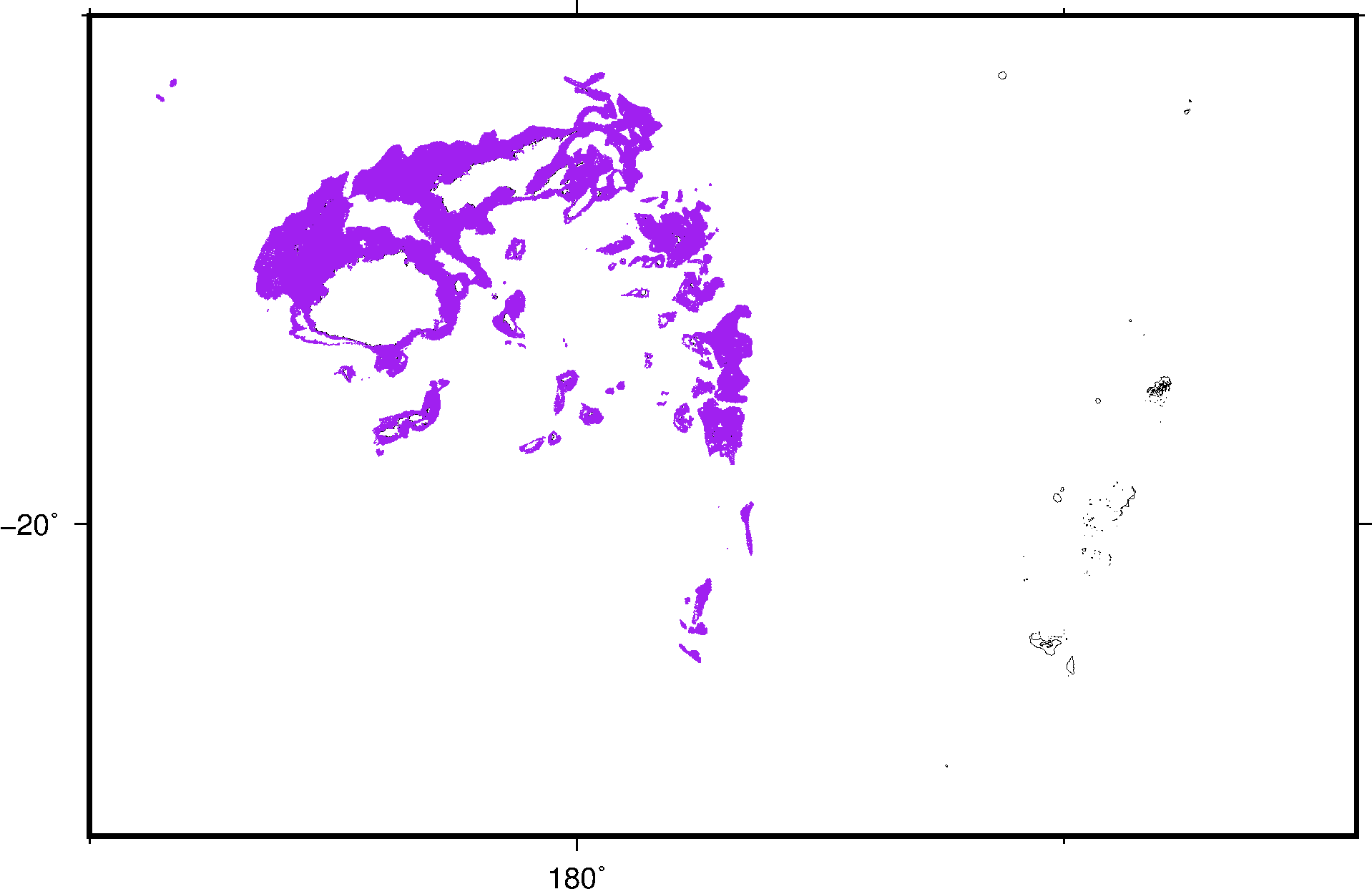

Supplement: S7 Gif — (GIF) [file pone.0234605.s009.gif]

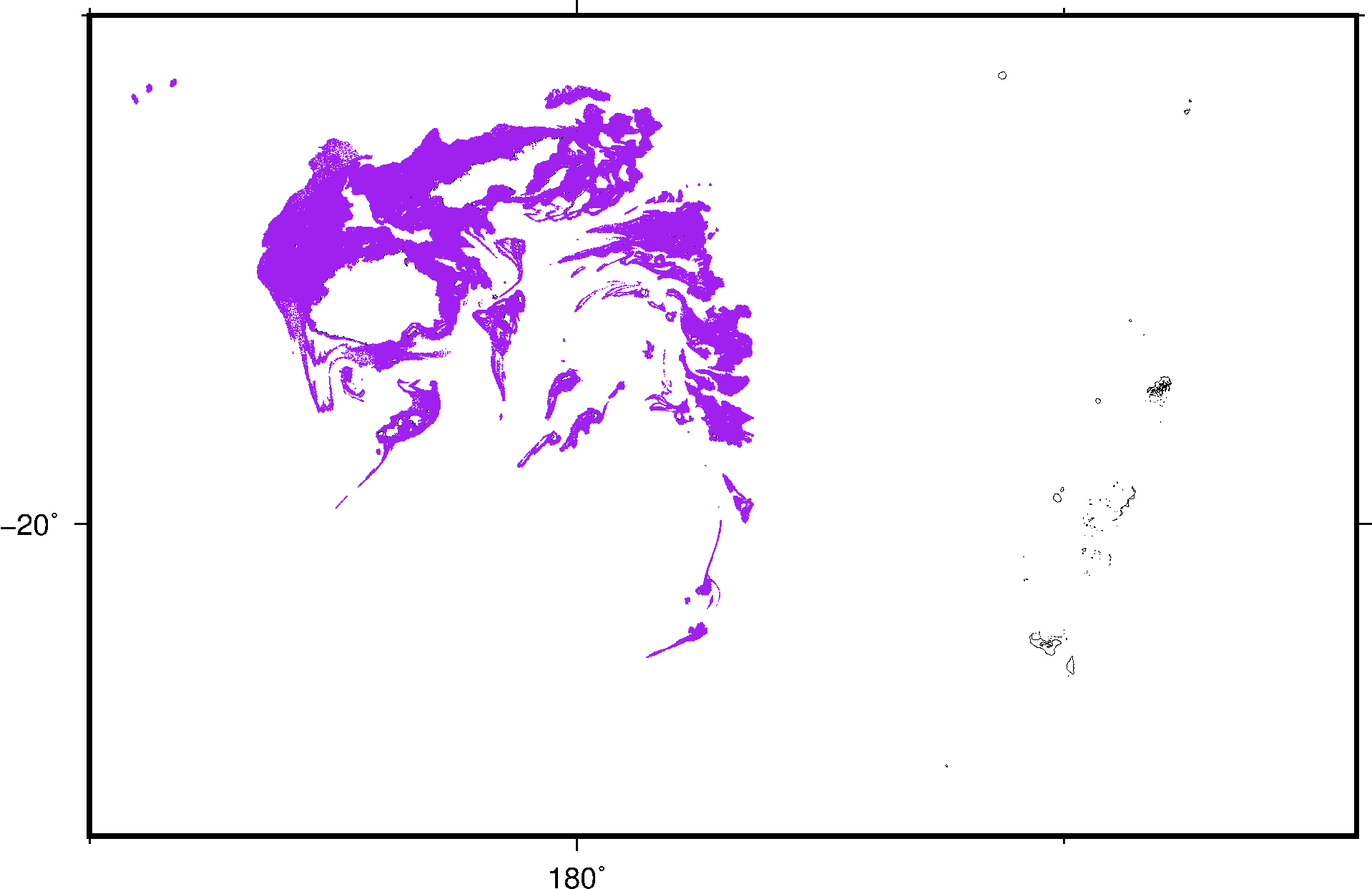

Supplement: S8 Gif — (GIF) [file pone.0234605.s010.gif]
