# Supplementary material for: Understanding marine larval dispersal in a broadcast-spawning invertebrate: A dispersal modelling approach for optimising spat collection of the Fijian black-lip pearl oyster Pinctada margaritifera
Source: PLoS One. 2020 Jun 18;15(6):e0234605. doi: 10.1371/journal.pone.0234605 (PMC7302709; doi:10.1371/journal.pone.0234605)
Supplement: S1 File — (DOCX) [file pone.0234605.s002.docx]

https://www.sciencedirect.com/science/article/pii/S2352513417300467
